# Ascorbic acid accumulates as a defense response to *Turnip mosaic virus* in resistant *Brassica rapa* cultivars

**DOI:** 10.1093/jxb/erw223

**Published:** 2016-06-02

**Authors:** Ayaka Fujiwara, Satoko Togawa, Takahiro Hikawa, Hideyuki Matsuura, Chikara Masuta, Tsuyoshi Inukai

**Affiliations:** Research Faculty of Agriculture, Hokkaido University, Sapporo 060-8589, Japan

**Keywords:** Ascorbate oxidase, ascorbate peroxidase, ascorbic acid, *Brassica rapa*, dehydroascorbate reductase, jasmonic acid, *Turnip mosaic virus*.

## Abstract

We initially observed that *Brassica rapa* cultivars containing the *Turnip mosaic virus* (TuMV) resistance gene, *Rnt1-1*, accumulated a high level of endogenous ascorbic acid (AS) and dehydroascobic acid (DHA) when infected with TuMV. We here hypothesized a possible contribution of an elevated level of AS+DHA (TAA) to the *Rnt1-1*-mediated resistance, and conducted a series of experiments using *B. rapa* and Arabidopsis plants. The application of l-galactose (the key substrate in AS synthesis) to a susceptible cultivar could increase the TAA level ~2-fold, and simultaneously lead to some degree of enhanced viral resistance. To confirm some positive correlation between TAA levels and viral resistance, we analyzed two Arabidopsis knockout mutants (*ao* and *vtc1*) in the AS pathways; the TAA levels were significantly increased and decreased in *ao* and *vtc1* plants, respectively. While the *ao* plants showed enhanced resistance to TuMV, *vtc1* plants were more susceptible than the control, supporting our hypothesis. When we analyzed the expression profiles of the genes involved in the AS pathways upon TuMV infection, we found that the observed TAA increase was mainly brought about by the reduction of AS oxidation and activation of AS recycling. We then investigated the secondary signals that regulate endogenous TAA levels in response to viral infection, and found that jasmonic acid (JA) might play an important role in TAA accumulation. In conclusion, we reason that the elevated TAA accumulation in *B. rapa* plants would be at least partly mediated by the JA-dependent signaling pathway and may significantly contribute to viral resistance.

## Highlight

Ascorbic acid accumulation is a potential resistance response to TuMV in *Brassica rapa* that is mediated via alterations in oxidation and recycling pathways.

## Introduction

l-Ascorbic acid (AS) is the major compound functioning in plant antioxidant systems and is present at millimolar levels inside plant cells (Zechmann, 2011). In contrast to mammals with only a single pathway for AS synthesis (Linster and Schaftingen, 2007), plants have four pathways to synthesize AS (Gallie, 2013*a*). The mannose/galactose pathway using d-glucose 6-phosphate as the starting substrate is the main AS synthesis pathway, and several alternative pathways have been described (Gallie, 2013*b*). In the recycling pathways, which also work to maintain the AS pool size, the oxidized forms of AS, monodehydroascorbic acid (MDHA) and dehydroascorbic acid (DHA), are reduced back to AS by MDHA reductase (MDHAR) and DHA reductase (DHAR), respectively (Gallie, 2013*b*).

In experiments using transgenic plants, overexpression of DHAR considerably increased the cellular AS levels, suggesting that AS accumulation was also limited by the recycling pathways in addition to the synthetic pathways (Gallie, 2013*b*). While AS enzymatically or non-enzymatically detoxifies reactive oxygen species (ROS) generated during aerobic metabolic processes such as photosynthesis or by abiotic stresses, this compound also regulates the cellular redox status as a major reductant (Foyer and Noctor, 2011). In addition to these functions, AS plays important roles in cell division, cell elongation, or the synthesis of phytohormones such as ethylene, abscisic acid (ABA), or gibberellic acid (GA) as a cofactor of the synthesizing enzymes (Gallie, 2013*a*).

In several plant species, total ascorbic acid (TAA: AS+DHA) accumulation followed ROS generation in response to abiotic stresses. For instance, after low temperature acclimation at 10 °C during 7 d, the TAA level in spinach (*Spinacia oleracea*) leaves was up to 41% higher than that in plants in normal conditions (Proietti *et al.*, 2009). Similar responses were observed in komatsuna (*Brassica rapa* var. *pervidiris*) leaves 8 d after the plants were transferred from 13–15 °C to 2–3 °C (Tamura, 1999). In leaves of *Arabidopsis thaliana* and tubers of potato (*Solanum tuberosum*), TAA accumulates after wounding, although the response patterns to the wound stress are quite different depending on the species and sampling sites of tissues (Suza *et al.*, 2010). TAA levels can also be increased by biotic stresses such as viral infection. In tomato (*Solanum lycopersicum*) fruits, TAA levels rose ~40% after infection with an attenuated strain of *Cucumber mosaic virus* (genus *Cucumovirus*, family *Bromoviridae*, order unassigned) (Tsuda *et al.*, 2005). While the induction mechanisms for TAA accumulation after such abiotic and biotic stresses are not yet understood well, jasmonic acid (JA), a plant hormone controlling response to biotic and abiotic stresses, has been reported to act as a signal for activation of AS synthesis or recycling in *A. thaliana* and tobacco (*Nicotiana tabacum*) (Sasaki-Sekimoto *et al.*, 2005; Wolucka *et al.*, 2005; Suza *et al.*, 2010). When Arabidopsis plants were treated with methyl jasmonate (MeJA), a significant increase in the mRNA level for four genes (the VTC1, VTC2, MDHR, and DHAR genes) has been found to be associated with an increase in TAA accumulation (Sasaki-Sekimoto *et al.*, 2005; Suza *et al.*, 2010). In tobacco suspension cells, the expression of the genes encoding GDP-mannnose 3',5'-epimerase and a putative gulono-1,4-lactone dehydrogenase/oxidase involved in the mannose/galactose pathway was up-regulated by MeJA treatment (Wolucka *et al.*, 2005).

*Turnip mosaic virus* (TuMV; genus *Potyvirus*, family *Potyviridae*, order unassigned), infects a wide range of hosts, including economically important *Brassica* species (Walsh and Jenner, 2002). Recently, we demonstrated using turnip (*B*. *rapa* subsp. *rapa*) plants that exogenous DHA and AS derivatives l(+)-ascorbic acid 2-sulfate disodium salt dehydrate (AS-SO_4_) and fat-soluble ascorbyl palmitate (AS-Pal) were useful as antiviral reagents against TuMV (Fujiwara *et al.*, 2013). In the present study, we extended our previous study to elucidate whether endogenous AS and/or DHA function as an antiviral compound against TuMV in *B*. *rapa* and whether TAA accumulation is induced in *B*. *rapa* plants in response to TuMV infection. Because resistant Chinese cabbage (*B. rapa* subsp. *pekinensis*) cultivars containing the *Rnt1-1* gene (Fujiwara *et al.*, 2011) could accumulate TAA at a high level when inoculated with an avirulent TuMV strain, we used this pathosystem to understand how the resistant *B. rapa* plants can regulate foliar TAA levels in response to TuMV.

## Materials and methods

### Plant materials and growth conditions

Nine turnip cultivars [CR Mochibana, CR Yukibana, Fuku-komachi, Kyo-senmai, Natsu-komachi, Wase-ohkabu (TAKII, Kyoto, Japan), Hakuro, Kyo-no-yuki, and Yukihime-kabu (TOHOKU SEED, Utsunomiya, Japan)] and two Chinese cabbage cultivars [Aki-masari (ATARIYA, Katori, Japan) and Ku-kai 65 (TAKII)] were used in this study. The *A. thaliana* (hereafter, Arabidopsis) ecotype Columbia (Col-0), the T-DNA insertion mutant for the *AO* gene (At5g21100), and the mutant for the *VTC1* gene (At2g39770) induced with ethyl methanesulfonate were also used (Conklin *et al.*, 1999; Yamamoto *et al.*, 2005). The *ao* mutant (SALK 108854) and the *vtc1* mutant (CS8326) were obtained from the Arabidopsis Biological Resource Center. Seeds of turnip and Chinese cabbage were soaked in water for 2–3 d under light to hasten germination, sown in peat pellets, Jiffy-7 (SAKATA, Yokohama, Japan), and grown in an MLR-350 growth chamber (SANYO, Tokyo) at 21 °C with a 12h photoperiod (150 μmol m^−2^ s^−1^). Seeds of Arabidopsis were sown directly into peat pellets, covered with a plastic wrap until germination, then grown in the same way as *B. rapa*.

### Virus inoculation, detection, and quantification

The TuMV strain that expresses yellow fluorescent protein (YFP)—TuMV-TuR1-YFP—and TuMV strain UK1 (Sánchez *et al.*, 1998) were kindly provided by Dr T. Natsuaki (Utsunomiya University, Japan) and Dr F. Ponz (INIA, Spain), respectively. The spontaneous mutant UK1 m2 from UK1 virulent to Aki-masari carrying *Rnt1-1* was obtained in our laboratory. These viruses had been maintained in the turnip cultivar Yukihime-kabu.

The second true leaves of *B. rapa* plants were dusted with carborundum and rub-inoculated with the inoculum 10 d after sowing. For Arabidopsis, four mature rosette leaves were inoculated 3 weeks after sowing in the same way as *B. rapa*. The extent of quantitative resistance to TuMV in plants was evaluated by counting the number of infection sites on the inoculated leaves using TuMV-TuR1-YFP. The infection sites were counted under blue light at 3–5 days post-inoculation (DPI). For the *ao* mutant of Arabidopsis, the regions infected by TuMV-TuR1-YFP on the non-inoculated upper leaves were harvested under blue light at 9 DPI, and viral levels were measured by ELISA using polyclonal antibodies against TuMV (Japan Plant Protection Association, Tokyo) according to the manufacturer’s instructions. Absorbance at 405nm was read using a microtiter plate reader ARVO MX 1420 (PerkinElmer Japan, Yokohama, Japan).

### Chemical treatments

l-Galactose, hydrogen peroxide (H_2_O_2_), and MeJA (Wako Pure Chemical Industries, Osaka, Japan), and salicylic acid (SA) and ABA (Sigma-Aldrich, St. Louis, MO, USA) were used in this study. A 10mM l-galactose solution was applied to *B. rapa* plants with a brush every day for 5 d before inoculation. The other chemicals were sprayed once on all aerial parts of *B. rapa* plants at various concentrations. At 24 hours post-treatment (HPT), the second true leaves were harvested for various analyses. For H_2_O_2_, the leaves were also sampled at 1, 6, and 12 HPT. In all experiments with chemical treatments, an experimental plot with water treatment was included as a control.

### Measurement of AS and DHA

Harvested leaves were immediately ground in 5 vols of 6% metaphosphoric acid with a mortar and pestle. The homogenate was centrifuged at 14 000rpm for 20min, and the supernatant was stored at –80 °C until analysis. The supernatant was diluted with 5% metaphosphoric acid 10-fold for AS and 5-fold for DHA based on the 2,4-diphenylhydrazine method (Bradley *et al.*, 1973).

### Gene expression analysis by quantitative RT-PCR

Total RNA was extracted from one half of the second true leaf on each plant using TRIzol reagent (Invitrogen, Tokyo, Japan) following the manufacturer’s instructions and treated with RNase-free DNase-I (Roche Diagnostics, Mannheim, Germany) to remove DNA contamination. The rest of the second true leaf was used to measure AS and DHA levels. The expression patterns of the genes involved in the AS synthesis, oxidation, and recycling pathways (Supplementary Table S1; Supplementary Fig. S1 at JXB online) were examined by the multiplex RT-PCR assay with the GenomeLAB GeXP Start Kit (Beckman Coulter, Fullerton, CA, USA) as described previously (Kim *et al.*, 2008). As internal standards, the TIP41-like protein gene, protein phosphatase 2A subunit A3 gene, and actin gene were chosen according to Chen *et al.* (2010) and Schuller and Ludwig-Müller (2006). The expression levels of the AS pathway genes were calculated as a ratio relative to the geometric mean of the three internal standard genes. The sequences of the primers used in this study are summarized in Supplementary Table S1.

### Enzyme activity assay

Ascorbate peroxidase (APX) activity was measured according to Nakano and Asada (1981). The second true leaves of *B. rapa* were harvested, weighed, and immediately homogenized in extraction buffer (50mM HEPES, 0.5mM AS, 0.5% Triton X-100, 20% sorbitol, plus 5% polyamide) at 10× the fresh mass of the sample. The homogenates were centrifuged for 15min at 14 000rpm and 4 °C; the supernatants were again centrifuged, and the second supernatants were used as enzyme extracts. The assay mixture consisted of 50mM potassium phosphate buffer (pH 7.0), 0.5mM AS, 0.1mM H_2_O_2_, 1mM EDTA and enzyme extract. APX activity was measured over 1min as a decrease in substrate concentration (0.5mM AS) using a spectrophotometer (U-2000, Hitachi High-Tech Fielding Corp., Tokyo, Japan) at 25 °C and 290nm (E=2.8mM^−1^ cm^−1^). One unit of enzyme activity was defined as the amount of enzyme catalyzing the oxidation of 1 μmol AS min^−1^ at 25 °C.

Ascorbate oxidase (AO) activity was measured according to Munyaka *et al.* (2010) using an enzyme extract prepared in the same way as APX but with 0.1M phosphate buffer (pH 5.6, 0.5mM EDTA, 0.75M NaCl, 0.5% Triton X-100, 20% sorbitol) containing 5% polyamide as the extraction buffer. The assay mixture consisted of 0.1M phosphate buffer (pH 5.6), 0.25mM AS, 0.5mM EDTA, and enzyme extract. AO activity was measured over 3min as the decrease in substrate concentration (0.25mM AS) using a spectrophotometer at 25 °C and 265nm (E=14mM^−1^ cm^−1^). One unit of enzyme activity was defined as the amount of enzyme catalyzing the oxidation of 1 μmol AS min^−1^ at 25 °C.

The enzyme extract for the MDHAR activity assay was prepared in the same way as for APX using 50mM phosphate buffer (pH 7.5, 0.1mM EDTA, 0.5% Triton X-100, 20% sorbitol) containing 5% polyamide as the extraction buffer. MDHAR activity was measured according to Hossain *et al.* (1984) in an assay mixture of 50mM TES buffer, 0.1mM NADH, 2.5mM AS, AO (1U ml^–1^) (Sigma-Aldrich), and enzyme extract. Activity was measured over 1min as the decrease in substrate concentration (0.1mM NADH) using a spectrophotometer at 25 °C and 340nm (E=6.2mM^−1^ cm^−1^). One unit of enzyme activity was defined as the amount of enzyme catalyzing the oxidation of 1 μmol of NADH min^−1^ at 25 °C.

DHAR activity was measured according to Hossain and Asada (1984) using an enzyme extract prepared in the same way with the same buffer as MDHAR. The assay mixture consisted of 50mM potassium phosphate buffer (pH 7.0), 0.5mM DHA, 5mM reduced glutathione, 1mM EDTA, and enzyme extract. DHAR activity was measured over 1min as the increase in AS concentration using a spectrophotometer at 25 °C and 265nm (E=14mM^−1^ cm^−1^). Correction was made for the rate of non-enzymatic reduction of DHA by reduced glutathione in the absence of enzyme extract. One unit of enzyme acivity was defined as the amount of enzyme catalyzing the reduction of 1 μmol of DHA min^−1^ at 25 °C.

Activities of all enzymes investigated in this study showed linear relationships with time and amount of extraction.

### Measurement of phytohormones

Harvested leaves were immediately weighed, frozen in liquid nitrogen, and stored at –80 °C until measurement using ultraperformance liquid chromatography–tandem mass spectrometry (UPLC-MSMS). The sample preparation and UPLC-MSMS were done as described previously (Matsuura *et al.*, 2009).

## Results

### Association between endogenous TAA level and the extent of quantitative resistance to TuMV

**Fig. 1.**
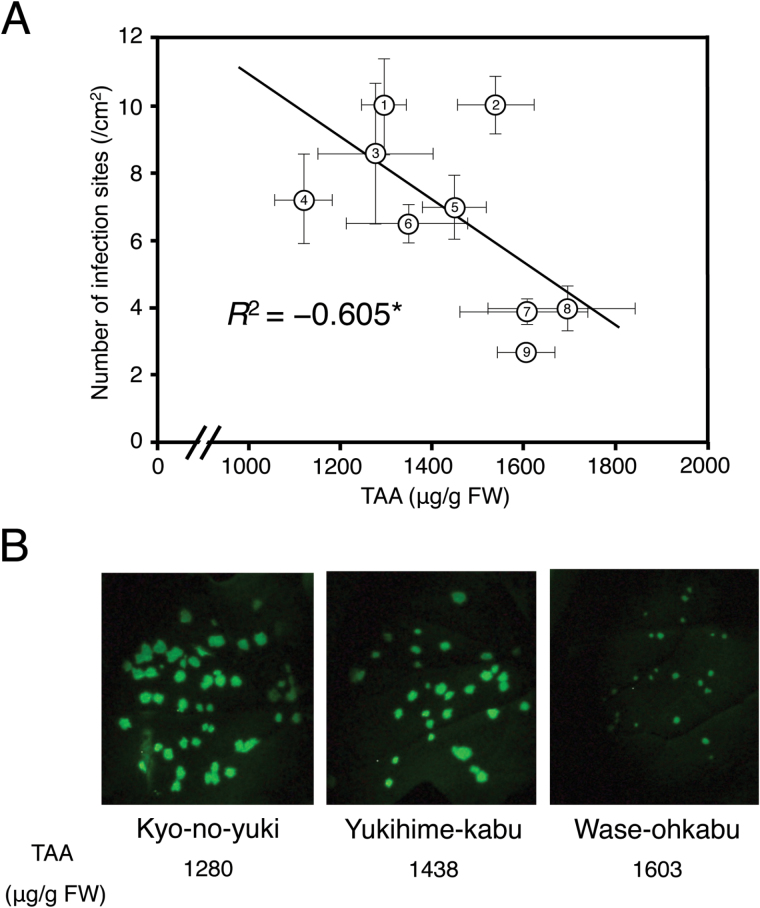
Correlation between the TAA (ascorbic acid and dehydroascorbic acid) content and *Turnip mosaic virus* (TuMV) resistance in turnip (*Brassica rapa* subsp. *rapa*). (A) Mean TAA content (*n*=3 plants) was positively correlated with the level of quantitative resistance to TuMV of nine turnip cultivars. TuMV resistance was evaluated based on the mean number of infection sites on the second true leaves (*n*=4–11 plants) 3 d after inoculation with the TuMV strain TuR1-YFP expressing yellow fluorescent protein (YFP). 1, Kyo-no-yuki; 2, Natsu-komachi; 3, CR Mochibana; 4, Fuku-komachi; 5, Yukihime-kabu; 6, Hakuro; 7, Wase-ohkabu; 8, Kyo-senmai, 9, CR Yukibana. Error bars indicate the SE for biological replicates. An asterisk showed that the correlation coefficient was significant at the 0.05 level. (B) Fluorescent spots indicating TuMV-TuR1-YFP infection on the second true leaves of three turnip cultivars showing the different levels of TAA. (This figure is available in colour at *JXB* online.)

To investigate whether endogenous TAA plays a role in inhibiting TuMV, we first examined the level of endogenous TAA in relation to the level of quantitative resistance to TuMV in the nine turnip cultivars. Among these cultivars, TAA levels varied from 1120.6 μg g^−1^ FW to 1675.3 μg g^−1^ FW (Fig. 1A). Using another set of plants grown under the same conditions, we counted the number of viral infection foci in the inoculated leaves infected with TuMV-TuR1-YFP, and found that the number of viral foci was negatively correlated with the TAA levels (Fig. 1A). Moreover, the spread of virus from the infection sites was also found to be slower on the turnip cultivars that contain higher levels of TAA (Fig. 1B).

**Fig. 2.**
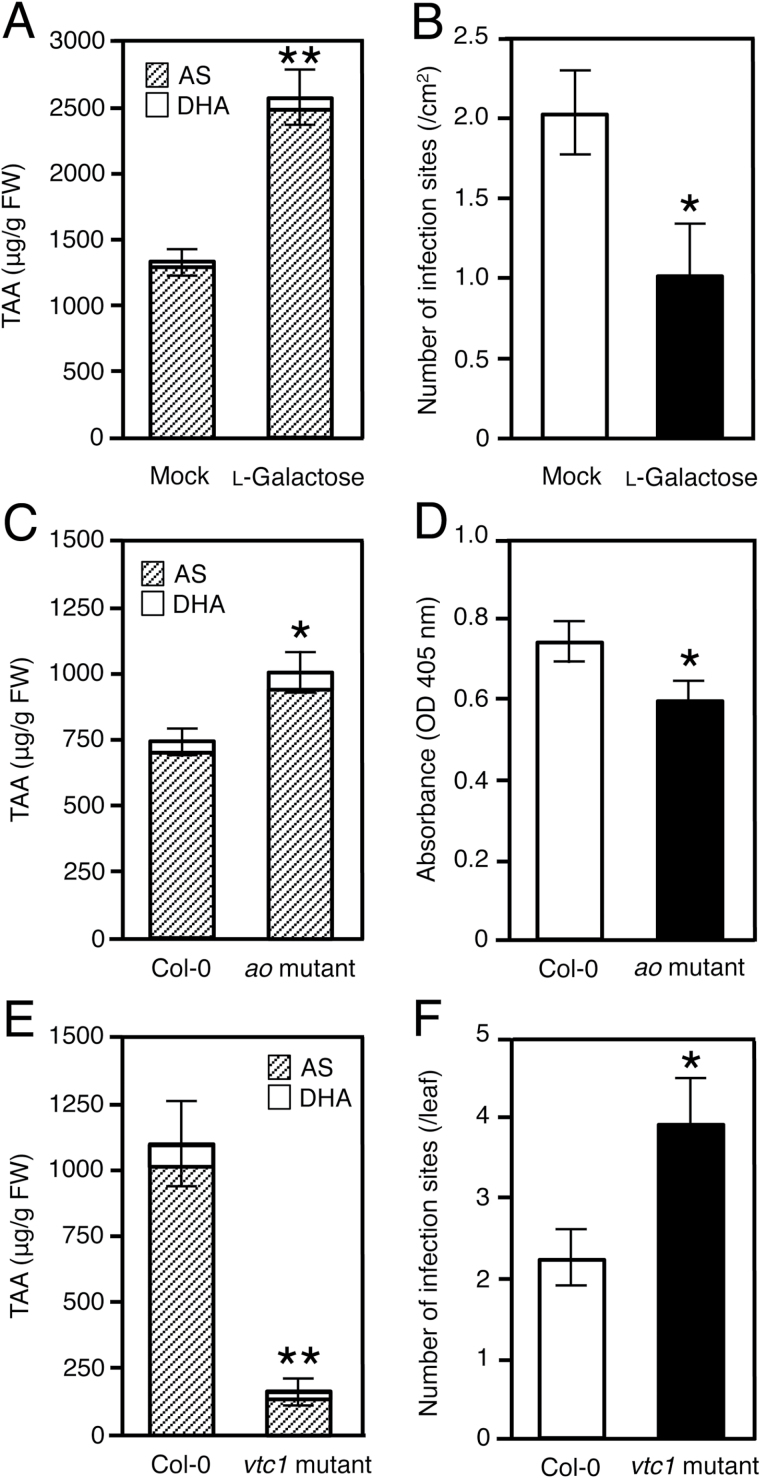
Effects of increased and decreased endogenous TAA [ascorbic acid (AS) and dehydroascorbic acid (DHA)] on *Turnip mosaic virus* (TuMV) infection in turnip (*Brassica rapa* subsp. *rapa*) and *Arabidopsis thaliana*. (A) TAA content in the second true leaves of turnip cultivar CR Mochibana treated with water (mock) or 10mM l-galactose. The l-galactose solution was brushed onto turnip plants daily for 5 d before inoculation. (B) Number of TuMV strain TuR1-YFP infection sites on the second true leaves of CR Mochibana treated with water (mock) or 10mM l-galactose. (C) TAA content in *A. thaliana* ecotype Columbia (Col-0) and the T-DNA insertion mutant of the ascorbate oxidase (*ao*) gene (At5g21100). (D) Viral accumulation of TuMV strain TuR1-YFP in non-inoculated upper leaves of wild-type Col-0 and the *ao* mutant. Viral levels were determined by measuring the absorbance at 405nm on a microtiter plate in an ELISA. (E) TAA content in Col-0 and the *vtc1* mutant. (F) Mean number of infection sites on four inoculated leaves of Col-0 and the *vtc1* mutant 5 d after inoculation with TuMV strain TuR1-YFP. Viral inoculation was carried out after treatment with 40h darkness. Error bars indicate the SE for 3–6 biological replicates. Asterisks represent significant differences determined by Student’s *t*-test (*P≤0.05, **P≤0.01).

To raise the level of endogenous TAA exogenously, we treated the turnip cultivar CR Mochibana with 10mM l-galactose, the substrate of l-galactose dehydrogenase in the mannose/galactose pathway of AS synthesis. A 2-fold increase in both TAA level (Fig. 2A) and TuMV resistance was found (Fig. 2B). Considering the possibility that l-galactose, one of the rare sugars, may have an elicitor activity like d-allose, another rare sugar (Kano *et al.*, 2010), we tested the activity of l-galactose as an elicitor of the expression of several defense genes in CR Mochibana plants treated with 10mM l-galactose. Because no significant changes in the expression of defense genes were observed (Supplementary Fig. S2), we concluded that the increase in TuMV resistance in the l-galactose-treated plants was due to the accumulation of TAA.

We next analyzed two Arabidopsis knockout mutants, which contain a mutated gene in the AS pathways. The Arabidopsis mutant *ao* has a T-DNA insertion in the AO gene (At5g21100) that results in a higher level of endogenous TAA than in the wild type (Yamamoto *et al.*, 2005). To evaluate the level of resistance to TuMV in the *ao* mutant, we inoculated seedlings 3 weeks after sowing with TuMV-TuR1-YFP and determined viral levels in infected tissues of non-inoculated upper leaves at 9 DPI. At the time of inoculation, the TAA level in the *ao* mutant was ~40% higher than that of the wild-type Col-0 (Fig. 2C). On the other hand, the viral level in the *ao* mutant had significantly decreased in comparison with the Col-0 control (Fig. 2D). We show a replicate experiment with a similar result in Supplementary Fig. S3.

The TAA-deficient mutant *vtc1* accumulated only 15% TAA of that in the wild-type Col-0 control (Fig. 2E). However, under normal light conditions, the SA-dependent stress resistance is activated due to the excess accumulation of ROS in the *vtc1* mutant (Mukherjee *et al.*, 2010). We therefore pre-treated the *vtc1* and the Col-0 plants by placing them in darkness for 40h before viral inoculation to cancel the ROS accumulation in the *vtc1* plants. After the dark treatment, those plants were inoculated with TuMV-TuR1-YFP and then incubated under low light conditions for 5 d. By comparing the number of viral infection foci in the inoculated leaves between the *vtc1* and the Col-0 plants, we found that the *vtc1* plants were more susceptible to TuMV than Col-0 (Fig. 2F). Taken together, these results suggest that an increase in endogenous TAA level is positively correlated with the viral resistance.

### TAA accumulation in response to TuMV in qualitatively resistant *B. rapa* cultivars

To clarify whether TAA accumulation was induced in response to TuMV infection in *B. rapa* plants as in tomato plants infected by an attenuated CMV (Tsuda *et al.*, 2005), the five Chinese cabbage and turnip cultivars with different responses to a severe strain of TuMV (UK1) were investigated. Chinese cabbage cultivars Aki-masari and Ku-kai 65 contain the TuMV resistance gene *Rnt1-1* and show qualitative resistance to UK1 (Supplementary Fig. S4). In these resistant cultivars, ~1.5- to 1.6-fold increases of TAA in the inoculated leaves were observed at 4 DPI (Supplementary Fig. S5). On the other hand, a susceptible Chinese cabbage cv. Yu-shun had a significant decrease in TAA in the inoculated leaves (Supplementary Figs S4, S5) while susceptible turnip cultivars CR Mochibana and Yukihime-kabu had no significant increase in TAA at 4 DPI (Supplementary Figs S4, S5). In these susceptible cultivars, leaf samples for TAA quantification were harvested before symptom development (Supplementary Fig. S4). TuMV strain TuR1-YFP, which causes mild symptoms, was also inoculated to CR Mochibana and Yukihime-kabu. In these cases, TAA quantification was carried out at 3 DPI; however, again no increase in TAA was observed (Supplementary Fig. S5). These results suggest that TAA accumulation is not induced in the compatible combinations between *B. rapa* and TuMV. We also examined whether the TAA level was elevated in Arabidopsis by TuMV infection. As in turnip, the TAA levels were not changed in either Col-0 or the *ao* mutant infected by TuMV-TuR1-YFP (Supplementary Fig. S6).

**Fig. 3.**
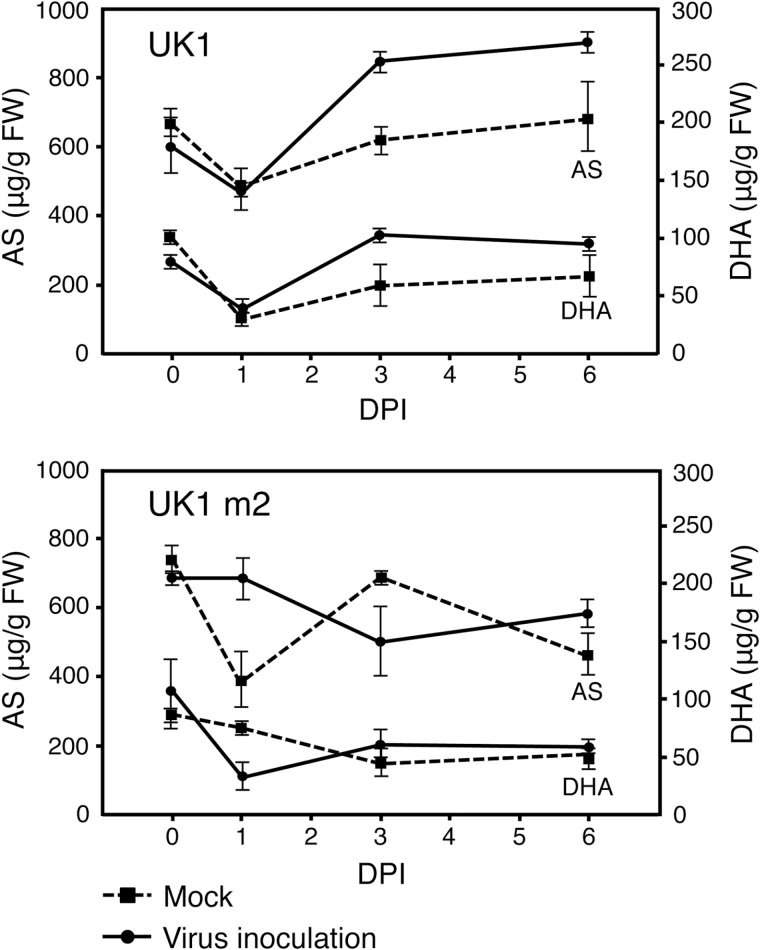
Change in content of ascorbic acid (AS) and dehydroascorbic acid (DHA) in Chinese cabbage (*Brassica rapa* subsp. *pekinensis*) cv. Aki-masari carrying *Rnt1-1* after inoculation with avirulent strain UK1 or virulent strain UK1 m2 of *Turnip mosaic virus*. Error bars indicate the SE for biological triplicates. DPI, days post-inoculation.

To confirm a link between TAA accumulation and *Rnt1-1* resistance, we compared the AS and DHA levels between the incompatible and compatible interactions of TuMV isolates on Aki-masari. In the incompatible combination of Aki-masari and TuMV-UK1, the AS and DHA levels in the inoculated leaves were, respectively, 1.4- and 1.9-fold significantly higher than in the mock-treated leaves at 3 DPI (Fig. 3). This high level of TAA was maintained even at 6 DPI. On the other hand, the TAA level did not increase during viral infection in the compatible combination of Aki-masari and TuMV-UK1-m2, a resistance-breaking mutant of UK1 (Fig. 3). These results indicate that there is a link between the TAA accumulation observed in Aki-masari and *Rnt1-1* resistance.

### Contribution of both suppression of AS oxidation and activation of DHA reduction to TAA accumulation in response to TuMV

**Fig. 4.**
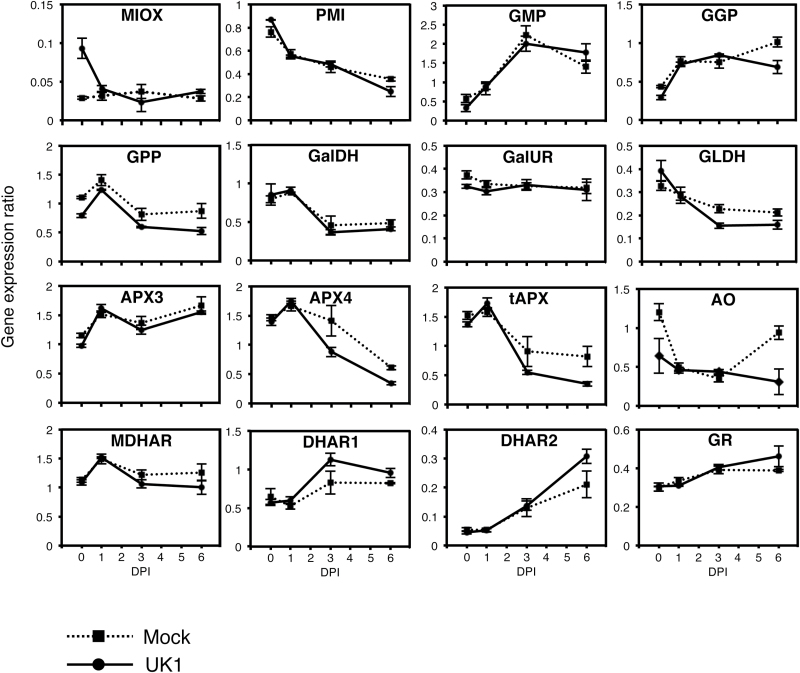
Expression profiles of genes for ascorbic acid synthesis, oxidation. and recycling in Chinese cabbage (*Brassica rapa* subsp. *pekinensis*) cv. Aki-masari inoculated with avirulent strain UK1 of *Turnip mosaic virus*. Transcript levels were determined with quantitative RT-PCR. Error bars indicate the SE for biological triplicates. See Supplementary Table S1 and Supplementary Fig. S1 for full names of each gene and their locations within the AS synthesis, oxidation, and recycling pathway diagrams. DPI, days post-inoculation.

**Fig. 5.**
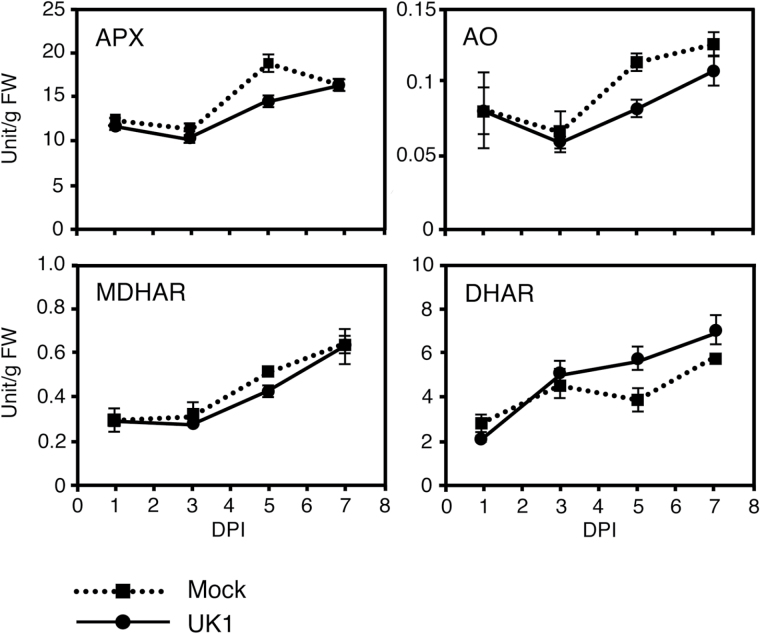
Enzyme activity profiles for ascorbic acid oxidation and recycling in Chinese cabbage (*Brassica rapa* subsp. *pekinensis*) cv. Aki-masari inoculated with avirulent strain UK1 of *Turnip mosaic virus*. Error bars indicate the SE of biological triplicates. APX, ascorbate peroxidase; AO, ascorbate oxidase; MDHAR, monodehydroascorbate reductase; DHAR, dehydroascorbate reductase; DPI, days post-inoculation.

The TAA pool size in plants is regulated by multiple and complicated pathways for synthesis, oxidation, and recycling (Supplementary Fig. S1). To elucidate which pathways are activated to increase the TAA level in Aki-masari after TuMV-UK1 infection, the expression level of the genes in each pathway was analyzed by quantitative RT-PCR. The results showed that none of the five genes involved in the mannose/galactose pathway was up-regulated by UK1 infection (Fig. 4). Neither the MIOX gene in the myo-inositol pathway nor the GalUR gene in the galacturonate pathaway was up-regulated by UK1 (Fig. 4). However, the expression levels of the genes involved in the AS oxidation and recycling pathways significantly changed. Among the three APX genes investigated, the APX4 and tAPX genes were down-regulated at 3 and 6 DPI (statistically supported at the 0.05 level) (Fig. 4). AO gene expression continued to decrease while that in Col-0 started to increase at 6 DPI (Fig. 4). The enzymatic activities of APX and AO slightly decreased at 3 DPI and then increased at 5 DPI (statistically supported at the 0.05 level) (Fig. 5). In the AS recycling pathways, the DHAR genes tended to increase (Fig. 4) and their enzymatic activities also increased during the observation period (statistically supported at the 0.05 level) (Fig. 5). In the susceptible cultivar Yukihime-kabu, the AS synthesis and recycling pathways were not activated by UK1 infection at the gene expression level at 4 DPI (Supplementary Fig. S7A). In the AS oxidation pathway, only the APX3 gene was down-regulated (Supplementary Fig. S7A). Similar results were obtained for another susceptible cultivar CR Mochibana infected by TuR1-YFP (Supplementary Fig. S7B). These results indicate that for the TAA accumulation in cultivars containing *Rnt1-1*, activation of the DHA-reducing pathway and suppression of the AS-oxidizing pathways were both important.

### Analysis of possible secondary signals to induce TAA accumulation in response to TuMV

To determine the signal transduction involved in the induction of TAA accumulation, we treated the second true leaves of Aki-masari with H_2_O_2_, a secondary messenger in plant defense response, or plant defense hormones such as SA, MeJA, and ABA. After treatment of Aki-masari with 25mM H_2_O_2_, the TAA level had slightly increased by 1 HPT and remained at nearly the same level until 24 HPT (Supplementary Fig. S8). The statistical significance of this increase in three independent experiments was confirmed (Table 1).

**Table 1.** Mean TAA (ascorbic acid and dehydroascorbic acid) content (µg g FW^−1^) in Chinese cabbage (*Brassica rapa* subsp. *pekinensis*) cv. Aki-masari 24h after treatment with 25mM H_2_O_2_, 50 µM salicylic acid (SA), or 10 µM methyl jasmonate (MeJA)

**Experiment**	**Mock**	**H**_**2**_ **O**_**2**_	**Mock**	**SA**	**Mock**	**MeJA**
1	694.4	738.3	783.8	859.1	818.1	955.3
2	838.9	897.3	721.6	626.1	636.6	685.0
3	418.8	515.2	781.8	718.2	804.4	890.0
*t*-value		4.231^*a*^		0.533		3.511^*a*^

Three biological replicates were used for each experiment.

^*a*^ The amount significantly differs from the mock treatment (*P≤*0.05; paired two-sample *t*-test).

On the other hand, SA did not induce TAA accumulation at any concentrations tested (1–100 μM) by 24 HPT (Table 1; Supplementary Fig. S8). When a higher concentration of SA (1mM) was tested, the TAA content instead decreased by 15% (data not shown). For MeJA, we observed an increase in TAA accumulation by 24 HPT (Supplementary Fig. S8). Because 10–50 µM MeJA treatment induced the highest levels of TAA, the 10 µM MeJA tests were repeated twice, and a statistically significant increase in the TAA level was confirmed (Table 1). The ABA treatment caused a significant decrease in the TAA level at 24 HPT (Supplementary Fig. S8).

**Fig. 6.**
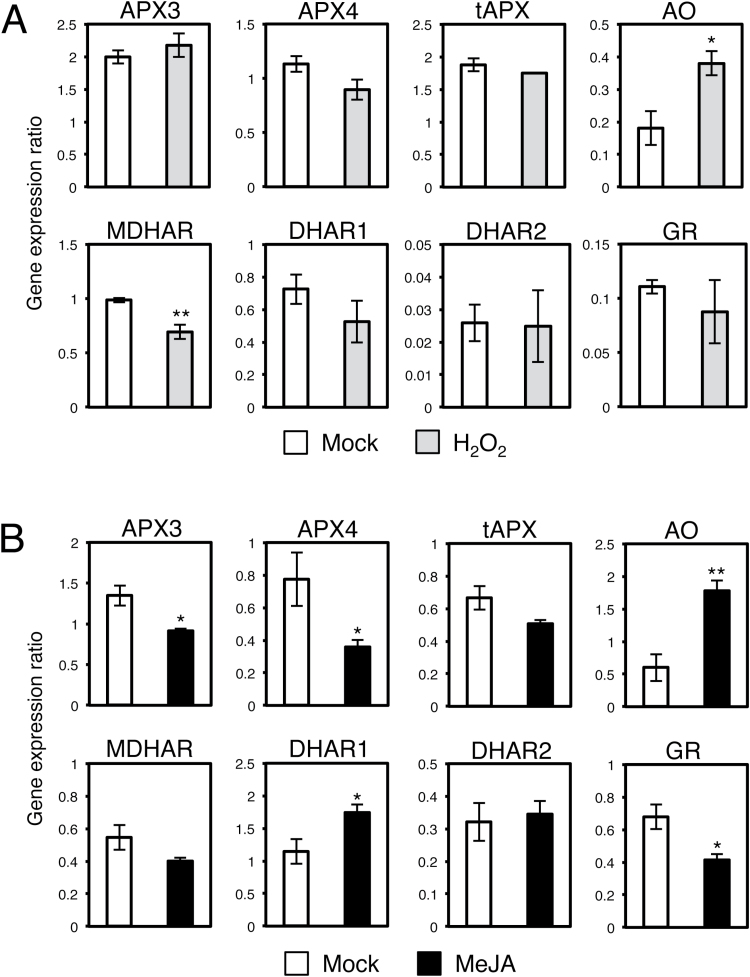
Expression profiles of genes for ascorbic acid oxidation and recycling in Chinese cabbage (*Brassica rapa* subsp. *pekinensis*) cv. Aki-masari after treatment with (A) 25mM H_2_O_2_ or (B) 50 µM methyl jasmonate (MeJA). Transcript levels were determined with quantitative RT-PCR 24h after treatment. Error bars indicate the SE for biological triplicates. Asterisks represent significant differences determined by Student’s *t*-test (*P≤0.05, ** *P*≤0.01). APX, ascorbate peroxidase; AO ascorbate oxidase; MDHAR, monodehydroascorbate reductase; DHAR, dehydroascorbate reductase; GR, glutathione reductase.

**Fig. 7.**
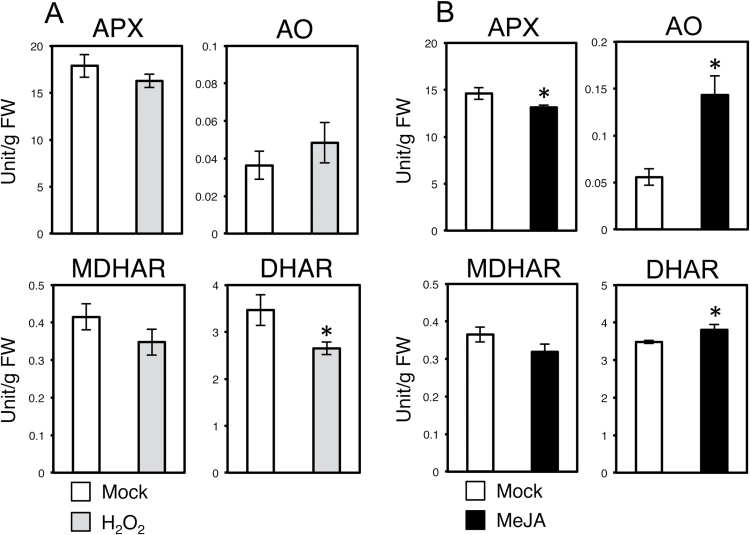
Enzyme activity profiles for ascorbic acid (AS) oxidation and recycling in Chinese cabbage (*Brassica rapa* subsp. *pekinensis*) cv. Aki-masari after treatment with (A) 25mM H_2_O_2_ and (B) 50 µM methyl jasmonate (MeJA). Enzyme activities were assayed 24h after treatment. Error bars indicate the SE for biological triplicates. Asterisks represent significant differences determined by Student’s *t*-test (*P≤0.05). APX, ascorbate peroxidase; AO ascorbate oxidase; MDHAR, monodehydroascorbate reductase; DHAR, dehydroascorbate reductase.

Because only H_2_O_2_ and MeJA induced TAA accumulation in Aki-masari, we next compared the expression patterns of the genes involved in AS oxidation and recycling between the UK1-inoculated and H_2_O_2_/MeJA-treated plants. After the H_2_O_2_ treatment, the AO gene was up-regulated, while the expression of MDHAR slightly decreased (Fig. 6A). When the enzymatic activities were measured, AO and MDHAR did not change significantly, but DHAR significantly decreased (Fig. 7A). Because the gene regulation patterns in the AS oxidation and recycling pathways after treatment with H_2_O_2_ differ from those after UK1 infection in Aki-masari, we concluded that ROS were unlikely to function as the signal for TAA accumulation.

On the other hand, for MeJA, the gene expression patterns in the AS oxidation and recycling pathways were significantly consistent with those induced after UK1 infection, except for the AO gene (Fig. 6B). The activity profiles for the enzymes involved in AS oxidation and recycling were also similar between MeJA treatment and UK1 inoculation, except for AO (Fig. 7B). These results thus suggest that JA may play an important role in TAA accumulation, but the only AO gene seems to be independently regulated.

**Fig. 8.**
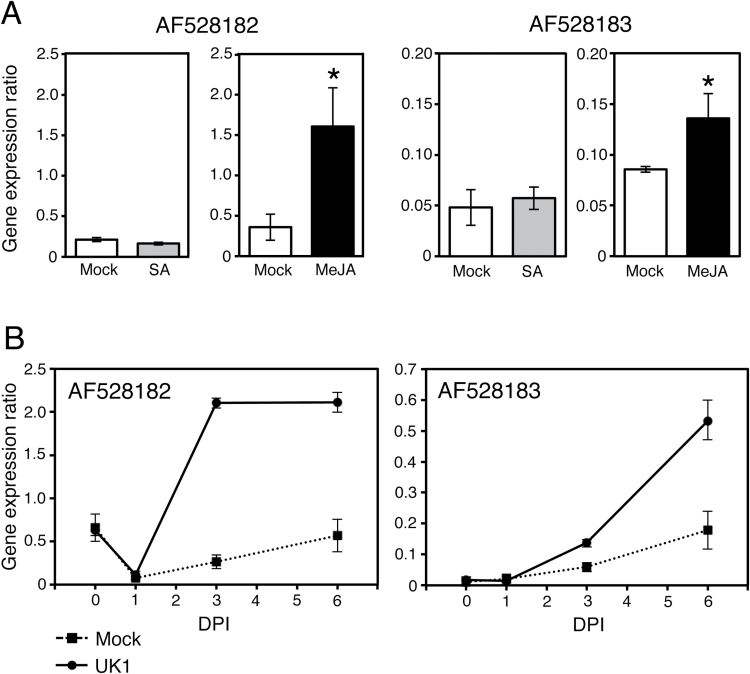
Expression of jasmonic acid-responsive genes in Chinese cabbage (*Brassica rapa* subsp. *pekinensis*) plants with *Rnt1-1* resistant to *Turnip mosaic virus*. (A) Responsiveness of genes AF528182 and AF528183 to 50 μM salicylic acid (SA) and 50 μM methyl jasmonate (MeJA). Transcript levels were determined with quantitative RT-PCR at 24h after inoculation. Asterisks represent significant differences determined by Student’s *t*-test (*P≤0.05). Error bars indicate the SE for biological triplicates. (B) Change in expression of AF528182 and AF528183 in Chinese cabbage cv. Aki-masari carrying *Rnt1-1* after inoculation with avirulent strain UK1 of *Turnip mosaic virus*. Transcript levels were determined with quantitative RT-PCR. Error bars indicate the SE for biological triplicates. DPI, days post-inoculation.

To confirm the involvement of the JA signaling pathway in TAA accumulation, we analyzed the expression pattern of the JA-responsive genes in the *Rnt1-1* plants inoculated with UK1. Although the *PDF1.2* gene has been generally used as a well-known marker for the JA signaling pathway, in *B. rapa* plants, the expression of *PDF.1.2* was not induced by the 50 μM MeJA treatment but rather was suppressed (Supplementary Fig. S9). Therefore, we selected the glucosyltransferase-like protein gene (AF528182) and putative metal-binding farnesylated protein gene (AF528183) (Park *et al.*, 2003) as JA signaling marker genes for *B. rapa*. Although Park *et al.* (2003) previously reported that the expression of these two genes was induced by 5mM SA but not by 1mM MeJA in *B. rapa*, in our analyses the expression of the two genes was induced by 50 µM MeJA treatment and mostly insensitive to 50 µM SA (Fig. 8A). This discrepancy would be due to the different MeJA concentrations used for each study. Because the physiologically active concentration of JA should be at the micromolar level (e.g. Van der Does *et al.*, 2013), we concluded that the two genes were specifically induced by MeJA at 50 µM. In Aki-masari inoculated with UK1, the expression of the two genes increased markedly, in parallel with the TAA increase (Figs 3, 8B), but AF528182 and AF528183 were not up-regulated in a susceptible cultivar CR Mochibana (Supplementary Fig. S10) that did not accumulate TAA after TuMV infection (Supplementary Fig. S5).

**Fig. 9.**
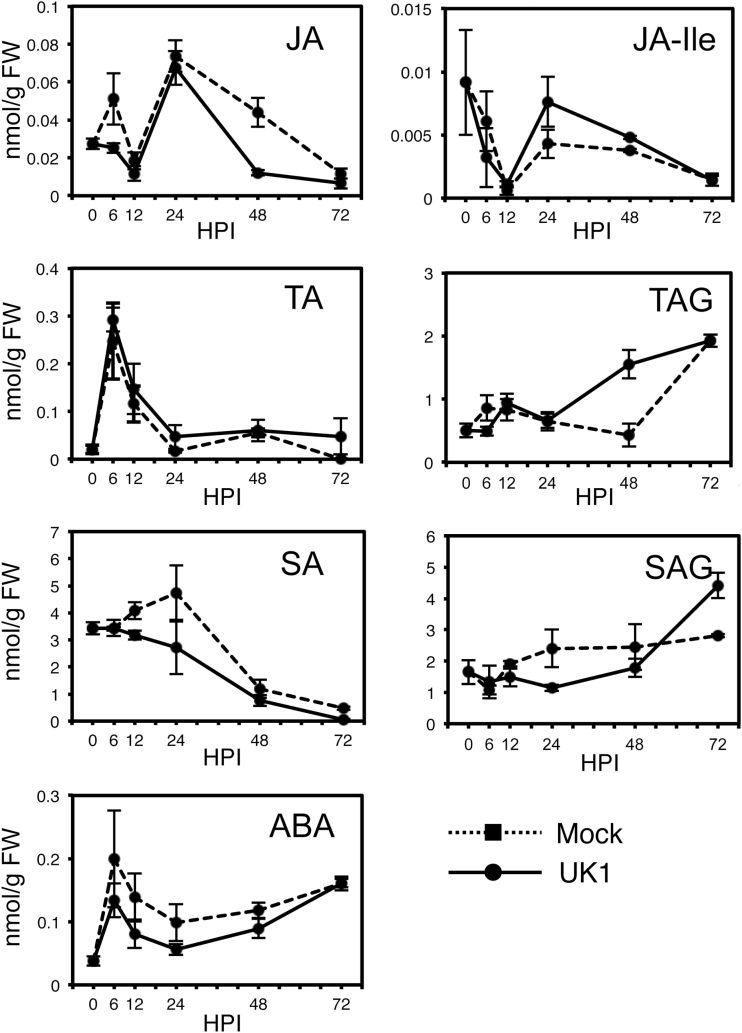
Change in levels of plant hormones in Chinese cabbage (*Brassica rapa* subsp. *pekinensis*) cv. Aki-masari inoculated with avirulent strain UK1 of *Turnip mosaic virus*. Error bars indicate the SE for biological triplicates. SA, salicylic acid; SAG, salicylic acid glucoside; JA, jasmonic acid; JA-Ile, jasmonic acid-isoleucine; TA, tuberonic acid; TAG, tuberonic acid glycoside; ABA, abscisic acid; HPI, hours post-inoculation.

Finally, we measured the levels of four endogenous plant hormones [jasmonates including JA-isoleucine (JA-Ile), SA, SA glucoside (SAG), and ABA]. Although JA had decreased by 48h after UK1 inoculation in Aki-masari compared with the mock-treated plants, JA-Ile, which is actually the active form of JA (Fonseca *et al.*, 2009), significantly increased between 24 and 48 hours post-inoculation (HPI; Fig. 9). The JA derivatives tuberonic acid (TA) and tuberonic acid glycoside (TAG) also greatly increased; TAG had increased >3-fold by 48 HPI. These increased levels of TA and TAG appear to be traces of the transient accumulation of JA because TA is directly synthesized from JA, and TA is then converted into TAG (Wakuta *et al.*, 2010). Considering these results together, we assume that JA transiently increased within 24h after UK1 inoculation and was then rapidly converted to JA-Ile, TA, or TAG. On the other hand, SA, SAG, and ABA tended to decrease during AS accumulation in Aki-masari after UK1 inoculation (Fig. 9). Because ABA seems to regulate AS accumulation negatively (Supplementary Fig. S8), the observed decline in ABA may have somehow contributed to TAA accumulation. Taken together, we reason that the JA-dependent signaling would be at least partly involved in the TAA accumulation leading to viral resistance.

## Discussion

### The genes involved in the AS oxidation and recycling pathways are responsible for the elevated level of TAA in a TuMV-resistant *B. rapa* cultivar

We showed that AS and DHA have an antiviral effect in both *B. rapa* and *A. thaliana* (Fujiwara *et al.*, 2013; this study); in *B. rapa*, the TAA level was elevated by 40–60% in a resistant cultivar Aki-masari containing *Rnt1-1*. The TAA increase in the *Rnt1-1* plants was ~300–500 µg g^–1^ FW (Fig. 3; Supplementary Fig, S5), equivalent to the difference in endogenous TAA level between quantitatively resistant and susceptible cultivars (Fig. 1A) and between the Arabidopsis *ao* mutant and the wild type (Fig. 2C). These results suggested that the TAA increase in the *Rnt1-1* plants had a certain effect to suppress viral infection. It is noteworthy that TAA behaves like two different antimicrobial molecules, a phytoanticipin, which accumulates at a certain level under normal conditions, and a phytoalexin, which is induced in response to pathogen attack.

The gene expression and enzyme activity profiles in Akimasari containing *Rnt1-1* clearly showed that TAA accumulation was a result of the suppression of AS oxidation pathways and the activation of the DHA reduction pathway rather than the acceleration of AS synthesis pathways. In the AS oxidation pathways, the suppression of both APX and AO seems to contribute to AS accumulation. While APX detoxifies ROS in cells (Gill and Tuteja, 2010), AO has been reported to be localized at the apoplast (Pignocchi and Foyer, 2003). Although the importance of AO in the maintenance of the TAA pool size in plant cells is not clear, the suppression of AO activity could increase the TAA level in cells, as shown in the *ao* mutant of Arabidopsis (Yamamoto *et al.*, 2005; this study). Because both APX and AO oxidize AS to produce MDHA, and MDHA was then spontaneously disproportionated to AS and DHA, the suppression of APX and AO and the activation of DHAR must lead to an increase in AS and a decrease in DHA. However, DHA actually increased in the *Rnt1-1* plants after UK1 inoculation (Fig. 3); this observation may be explained by a slight decrease in MDHAR activity (Figs 4, 5).

### Possible involvement of JA in the regulation of TAA levels in *B. rapa*

As shown in Figs 4 and 5, in the *Rnt1-1* plants infected with TuMV, APX and AO decreased and DHAR increased at the level of both transcript and enzyme. Because in the TuMV-infected tissues JA derivatives seemed to be significantly increased (Fig. 9), we inferred that JA could regulate the key genes responsible for elevation of the TAA level in *B. rapa*. When Aki-masari plants were exogenously treated with MeJA, we confirmed that APX decreased and DHAR increased at the level of both transcript and enzyme (Figs 6, 7), similar to the TuMV infection. The only exception was the *AO* gene, which was decreased by TuMV and increased by JA. Because in tobacco, SA, an antagonist of JA, has been demonstrated to decrease *AO* expression (Pignocchi *et al.*, 2003), the *AO* gene may be regulated in a complex balance between the SA and JA levels. Alternatively, the *AO* gene may be regulated independently of both JA and SA in *B. rapa*.

Involvement of JA in the AS pathways has been reported in several plant species (Sasaki-Sekimoto *et al.*, 2005; Wolucka *et al.*, 2005; Suza *et al.*, 2010). For example, 50 μM MeJA treatment to Arabidopsis rosette leaves resulted in just a 7% increase in TAA accumulation (Suza *et al.*, 2010). When very young plants (10 d after sowing) were treated with 200 μM MeJA, a 2.8-fold TAA increase was detected (Sasaki-Sekimoto *et al.*, 2005). The TAA levels elevated by JA seem to be dependent on the developmental stage of the plants used; the younger the plants, the higher the levels of TAA which tend to accumulate. In those experiments, the VTC1 (GMP), VTC2 (GGP), MDHAR, and DHAR genes were all reported to be up-regulated ~2- to 4-fold (Sasaki-Sekimoto *et al.*, 2005; Suza *et al.*, 2010). However, in this study, we could not confirm such gene activation of the *VTC1* and *VTC2* genes in *B. rapa* plants when treated with 10 μM MeJA (Supplementary Fig. S11). Taken together, we consider that although the TAA accumulation is generally regulated through the JA signaling pathway in plants belonging to *Brassicaceae*, the manner of regulation for each gene involved in the AS pathways may not necessarily be the same among plant species.

### Association of TAA accumulation mediated by JA with the *Rnt1-1*-induced TuMV resistance in *B. rapa*

There must be a series of host responses for the *Rnt1-1*-induced resistance at an initial stage of infection. On the other hand, because TAA gradually accumulates to the maximum level until 3–6 DPI, the TAA-mediated resistance would be effective at a late stage perhaps to inhibit viral spread but not infection. When we analyzed the expression levels of the marker genes (AF528182 and AF528183) for JA in TuMV-infected *B. rapa* plants, their expression was induced in *Rnt1-1* plants (Fig. 8) but not in susceptible plants Supplementary Fig. S10). In addition, in the *Rnt1-1* plants infected by TuMV, endogenous levels of JA derivatives were increased (Fig. 9). These results suggest that JA must be at least one of the secondary signals for the *Rnt1-1*-induced resistance. Because exogenous application of MeJA resulted in activation of some important genes in the AS pathways, we believe that JA accumulation is induced by TuMV in *Rnt1-1* and may subsequently cause the activation of the AS pathways.

### Endogenous TAA has multifunctional roles in response to various pathogens

Although we demonstrated that the TAA level was positively correlated with the extent of viral resistance, negative correlations also exist. For example, an AS deficiency can increase resistance to other pathogens such as *Pseudomonas syringae* and *Peronospora parasitica* in Arabidopsis (Barth *et al.*, 2004). In the *vtc* mutants in which the AS levels decreased to ≤40% of the wild-type level, both H_2_O_2_ and SA levels are increased (Mukherjee *et al.*, 2010), inducing some defense responses. On the other hand, an excess amount of AS may suppress the induction of defense responses mediated by H_2_O_2_, making plants more susceptible to pathogens. Therefore, endogenous levels of TAA must be, we believe, strictly regulated so that plants can properly activate defense depending on the pathogen species.

AS has been reported to work not only against viruses but also against other pathogens. For instance, treatment of the rice blast fungus *Magnaporthe oryzae* with AS decreased the percentage of normal appressorium formation because ROS, which function as signal molecules for appressorium formation, are degraded by AS (Egan *et al.*, 2007). AS was also reported to be deleterious to hyphal development of *Alternaria brassicicola* (Botanga *et al.*, 2012). Interestingly, the accumulation of sakuranetin, a phytoalexin identified from blast resistance rice cultivars, was induced by JA; this JA-dependent induction was enhanced by co-treatment with a high concentration of AS (Tamogami *et al.*, 1997). These observations may indicate that AS has both direct and indirect effects in defense responses to fungal pathogens as well. In conclusion, we believe that AS can work as a standing weapon against a wide range of pathogens including viruses and fungi.

## Supplementary data

Supplementary data are available at *JXB* online.

Table S1. List of primers used for gene expression analyses.

Figure S1. Proposed pathways for AS biosynthesis, oxidation, and recycling in plants.

Figure S2. Expression level of defense genes in turnip cv. CR Mochibana after treatment with 10mM l-galactose.

Figure S3. Viral accumulation of TuMV strain TuR1-YFP in non-inoculated upper leaves of Arabidopsis ecotype Col-0 and the *ao* mutant.

Figure S4. Symptoms developed on leaves of the five Chinese cabbage and turnip cultivars with different alleles at the *Rnt1* locus with the TuMV strain UK1.

Figure S5. Mean TAA content in the five Chinese cabbage and turnip cultivars carrying different alleles at the *Rnt1* locus after inoculation with TuMV strains UK1 and TuR1-YFP.

Figure S6. Mean TAA content in the Arabidopsis ecotype Col-0 and the *ao* mutant after inoculation with TuMV strain TuR1-YFP.

Figure S7. Expression profiles determined with quantitative RT-PCR for genes for AS synthesis, oxidation, and recycling in turnip ccultivrs.

Figure S8. Mean TAA content in second true leaves of Chinese cabbage cv. Aki-masari at 24h after treatment with H_2_O_2_, SA, MeJA, or ABA.

Figure S9. Expression change of *PDF1.2* in Chinese cabbage cv. Aki-masari after treatment with 50 µM MeJA.

Figure S10. Expression levels of the AF528182 and AF528183 genes in turnip cv. CR Mochibana by infection of TuMV strain TuR1-YFP.

Figure S11. Expression levels of the *VTC1* and *VTC2* genes in Chinese cabbage cv. Aki-masari after treatment with 50 µM MeJA.

Supplementary Data
