# Supplementary material for: Ascorbic acid accumulates as a defense response to Turnip mosaic virus in resistant Brassica rapa cultivars
Source: J Exp Bot. 2016 Jun 2;67(14):4391–402. doi: 10.1093/jxb/erw223 (PMC5301938; doi:10.1093/jxb/erw223)
Supplement: Supplementary Data [file supp_67_14_4391__index.html]

Ascorbic acid accumulates as a defense response to Turnip mosaic virus in resistant Brassica rapa cultivars — Ascorbic acid accumulates as a defense response to Turnip mosaic virus in resistant Brassica rapa cultivars — Supplementary Data 

# Ascorbic acid accumulates as a defense response to *Turnip mosaic virus* in resistant *Brassica rapa* cultivars

## Supplementary Data

Data files

- supplementary\_table\_S1\_Figures\_S1\_S11.pdf - Supplementary Data
