# Supplementary material for: Ascorbic acid accumulates as a defense response to Turnip mosaic virus in resistant Brassica rapa cultivars
Source: J Exp Bot. 2016 Jun 2;67(14):4391–402. doi: 10.1093/jxb/erw223 (PMC5301938; doi:10.1093/jxb/erw223)
Supplement: Supplementary Data [file supp_erw223_supplementary_table_S1_Figures_S1_S11.pdf]

Table S1 *Brassica rapa* genes included in expression analysis

			A. thaliana	*B. rapa*				
	Gene	Encoded enzyme	Gene ID	GenBank acc. no.	Gene ID	Forward primer (5' to 3')	Reverse primer (5' to 3')	References
Internal control	PP2A	---	---	EX054539	Bra025162	AGGTGACACTATAGAATAATTTGATGCAATATTCTGAACTCTCA	GTACGACTCACTATAGGGAACAACTTAGGCTGTTTGCATGA	Chen et al. (2010) Anal Biochem 405:138–140
	TIP41	---	---	EX117271	Bra011516	AGGTGACACTATAGAATAACCTGAGGGGGAAGCTAGTC	GTACGACTCACTATAGGGACTCCATTGTCAGCCAGTTCA	Chen et al. (2010) Anal Biochem 405:138–140
	Actin	---	---	EX139908	Bra037560	AGGTGACACTATAGAATAACATTCCAGCAGATGTGGATCTC	GTACGACTCACTATAGGGAAACCCAGAGAGTTTTGTCACAC	Schuller and Ludwig-Müller (2006) New Phytologist 171: 145–157
AsA synthesis	MIOX	Myoinositol oxygenase	At4g26260	EX132809	Bra041014	AGGTGACACTATAGAATAGCTGGCTATGAAGTAATTAGTCCTG	GTACGACTCACTATAGGGAACTCGCTATGGATGATTGTGA	
	PMI	Phosphomannose isomerase	At1g42550	ES934679	Bra021430	AGGTGACACTATAGAATAGGGAACTCGCAATTGAGTC	GTACGACTCACTATAGGGACACTGGAGCCATCAGCAT	
	GMP	GDP-D-mannose pyrophosphorylase	At2g39770	EX141191	Bra005014	AGGTGACACTATAGAATATTTTCATCTTTGTGCTTTCGG	GTACGACTCACTATAGGGATTACAAAACACCAAATGACTTTTAAC	
	GGP	GDP-L-galactose phosphorylase	At4g26850	EX092676	Bra010424	AGGTGACACTATAGAATATTCTCCGGCGAAAATTGG	GTACGACTCACTATAGGGATGCGGCAAAGGGTTAGTCT	
	GPP	L-galactose-1-phosphate phosphatase	At3g02870	EX061908	Bra040607	AGGTGACACTATAGAATAAAGCTGGACAGGTGATTCGT	GTACGACTCACTATAGGGAACCATTCGCAGCTGTTGTTT	
	GalDH	L-galactose dehydrogenase	At4g33670	EX133611	Bra036989	AGGTGACACTATAGAATATGGGAAACACAGGTCTCAAA	GTACGACTCACTATAGGGATTAATGCCTTGTCGGAACG	
	GLDH	L-galactono-1,4-lactone dehydrogenase	At3g47930	ES933110	Bra018100	AGGTGACACTATAGAATAATACGCAGCACTGGCTCTCT	GTACGACTCACTATAGGGAGTCTCCGGCTGGTTAAAGTTC	
	GalUR	D-galacturonate reductase	---	EX083199	Bra027828	AGGTGACACTATAGAATATGAAGTGGAGGAATGCAAGA	GTACGACTCACTATAGGGAGCAGTCAAAACAATGCCCTT	Agius et al. (2003) Nat Biotechnol 21:177-181
AsA oxidation	APX3	Ascorbate peroxidase 3	At4g35000	EX121179	Bra013053	AGGTGACACTATAGAATACAACGTCTACAGTCACTATGGC	GTACGACTCACTATAGGGAGAGAAGAGAAGGGGAATGCGT	
	APX4	Ascorbate peroxidase 4	At4g09010	DY010066	Bra000663	AGGTGACACTATAGAATACTGCATTGACAAAGCTAAGTGG	GTACGACTCACTATAGGGATTGTTGTCACGCTTATGCTCA	
	tAPX	Thylakoidal ascorbate peroxidase	At1g77490	EX054285	Bra015668	AGGTGACACTATAGAATATTCCACCCAAAAGAAAGAGC	GTACGACTCACTATAGGGAACAAGAGGACCAAAACGCTG	
	AO	Ascorbate oxidase	At5g21105	XM_009128252	Bra020145	AGGTGACACTATAGAATAGGGAAGAAAGAGCCATTGA	GTACGACTCACTATAGGGATGTTGGTAACGATGTGCTGC	
AsA recycling	MDHAR	Monodehydroascobate reductase	At3g27820	EX100231	Bra025303	AGGTGACACTATAGAATAGGTGAGTCAGCATATAGTTCCAG	GTACGACTCACTATAGGGAGAGGCTTCTCCATCATCACCA	
	DHAR1	Dehydroascobate reductase	At1g19570	EX046186	Bra008662	AGGTGACACTATAGAATAACTAGTTCCATCTGCTCCGC	GTACGACTCACTATAGGGAAGCATAATAGATTGTTTGGTTAGAACA	
	DHAR2	Dehydroascobate reductase	At1g75270	EE521888	Bra008188	AGGTGACACTATAGAATACGTGCGAAATCTGAAGATCG	GTACGACTCACTATAGGGACGGAGACGTTAGCGAGATGA	
	GR	Glutathione reductase	---	AF008441	Bra015006	AGGTGACACTATAGAATACACACACCATCTTAGAGAGTTTGG	GTACGACTCACTATAGGGATCATAGTGAGTCTCGGTTGTAGC	Lee et al. (1998) Biochim Biophys Acta 1395:309-314
Defense	PR1	---	At2g14610	DN237938	Bra013123	AGGTGACACTATAGAATAACGTGCAATGGAGAATGCCT	GTACGACTCACTATAGGGATTGCCCCGAGGATCATAGTT	
	PDF1.2b	---	At2g26020	EX083692	Bra015809	AGGTGACACTATAGAATATCTACCAAAATCATGGGTGCT	GTACGACTCACTATAGGGACTGCTACATGTCATGATGTCACTC	
	RDR1	---	At1g14790	EX127661	Bra026187	AGGTGACACTATAGAATAAATCTATTGGAAGGGCGGTT	GTACGACTCACTATAGGGATCACATGATACCAAGCCGAG	
	RDR6	---	At3g49500	EV015278	Bra029957	AGGTGACACTATAGAATACCCTGAAGAAAGAGCTGAGG	GTACGACTCACTATAGGGAAAGCAAAGCTCAGCATCACC	
JA responsive	AF528182	Glucosyltransferase-like protein		AF528182		AGGTGACACTATAGAATACCTGACGTCATCGAGCTTCT	GTACGACTCACTATAGGGAATCACACAAGCAGCAGTTGG	Park et al. (2003) Plant Cell Rep 21: 1027–1034
	AF528183	Putative metal-binding farnesylated protein		AF528183		AGGTGACACTATAGAATAGCAGAGCACTGGGAAAAAGA	GTACGACTCACTATAGGGATCCGACTTCCTCACAAAACC	Park et al. (2003) Plant Cell Rep 21: 1027–1034

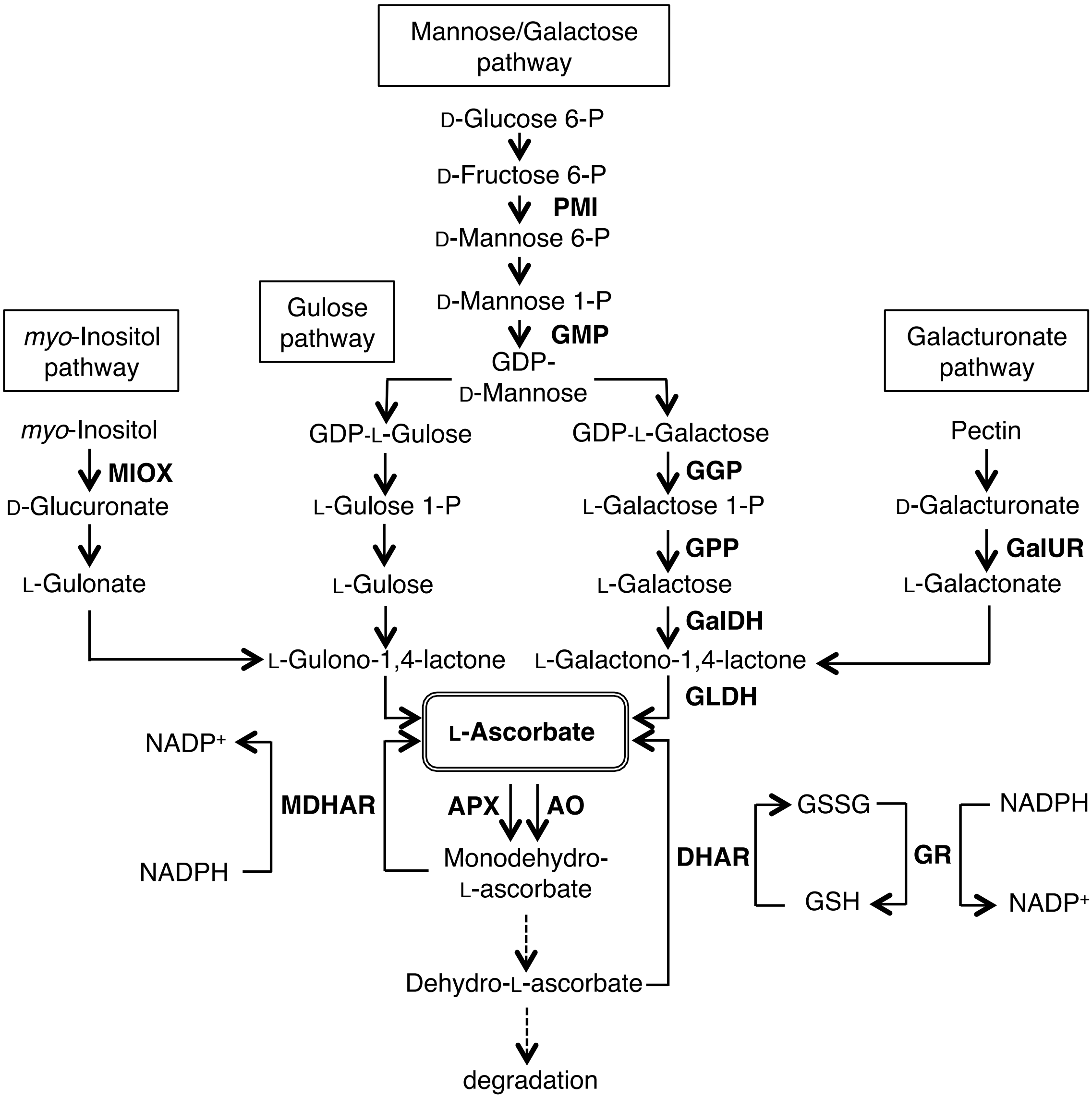

Supplementary Figure S1

Proposed pathways for ascorbic acid biosynthesis, oxidation and recycling in plants. Arrows indicate a series of enzymatic reactions on each pathway, and the genes encoding each enzyme studied in this study are detailed on the pathways.

PMI, phosphomannose isomerase
GMP, GDP-D-mannose pyrophosphorylase
GGP, GDP-L-galactose phosphorylase
GPP, L-galactose-1-phosphate phosphatase
GalDH, L-galactose dehydrogenase
GLDH, L-galactono-1,4-lactone dehydrogenase
MIOX, myoinositol oxygenase
GalUR, D-galacturonate reductase
APX, ascorbate peroxidase
AO, ascorbate oxidase
MDHAR, monodehydroascorbate reductase
DHAR, dehydroascorbate reductase
GR, glutathione reductase

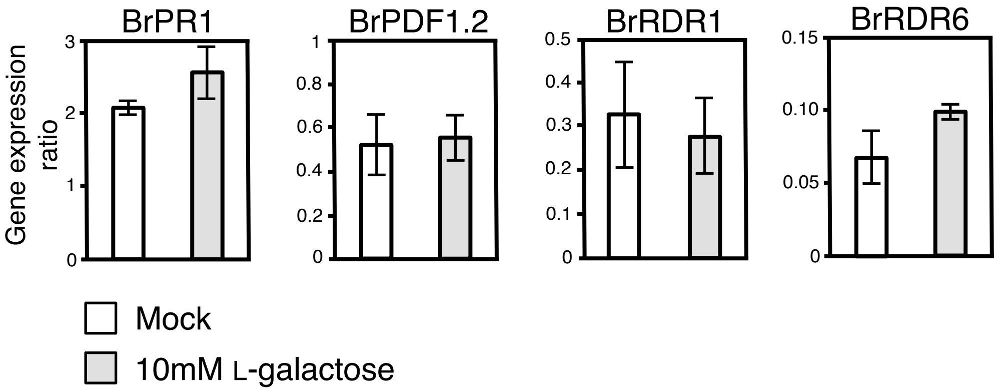

Supplementary Figure S2

Expression level of defense genes in turnip (*Brassica rapa* subsp. *rapa*) cv. CR Mochibana after treatment with 10 mM L-galactose. Transcript levels were determined with quantitative RT-PCR 24 h after treatment. Error bars indicate standard error for biological triplicates. PR, pathogenesis-related protein; PDF, plant defensin; RDR, RNA-dependent RNA polymerase.

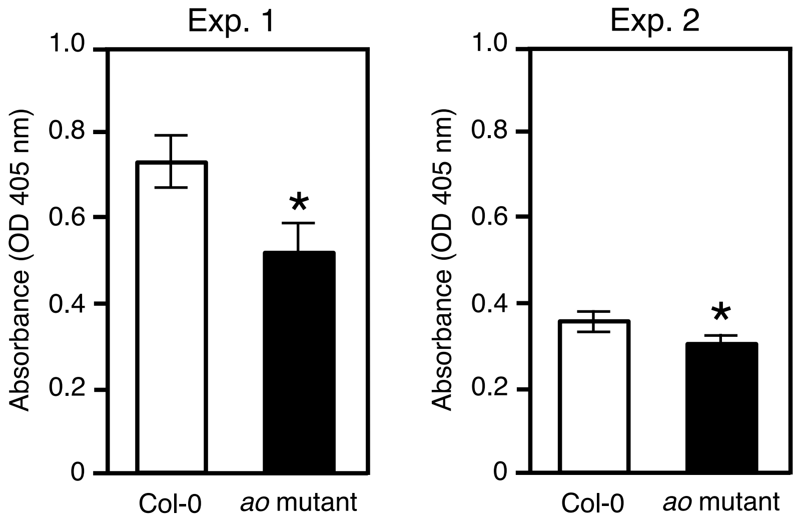

Supplementary Figure S3

Viral accumulation of *Turnip mosaic virus* strain TuR1-YFP in non-inoculated upper leaves of an *Arabidopsis thaliana* ecotype Columbia (Col-0) and the *ao* mutant. Error bars indicate standard error for three to four biological replicates. Asterisks represent significant differences determined by Student's *t*-test (*$P \leq 0.05$).

Supplementary Figure S4

Symptoms on inoculated and uninoculated upper leaves of the five Chinese cabbage (*Brassica rapa* subsp. *pekinensis*) and turnip (*Brassica rapa* subsp. *rapa*) cultivars with different alleles at the *Rnt1* locus. Second true leaves of each plant were inoculated with *Turnip mosaic virus* (TuMV) strain UK1. Chinese cabbage cvs. Aki-masari and Ku-kai 65 with resistance gene *Rnt1-1* did not develop lesions. In Chinese cabbage cv. Yu-shun carrying *rnt1-2*, necrotic lesions had developed by 5 days post inoculation (DPI) and expanded more by 8 DPI. At 14 DPI, the plants were systemically infected. Turnip cv. CR Mochibana carrying *rnt1-3* did not have any visible symptoms at 4 DPI, but chlorotic and necrotic spots had appeared on inoculated leaves by 8 DPI; by 14 DPI, mosaic symptoms were observed on uninoculated upper leaves. Turnip cv. Yukihime-kabu carrying *rnt1-3* had symptoms similar to those on CR mochibana, but yellowing of inoculated leaves developed earlier.

Inoculated leaves

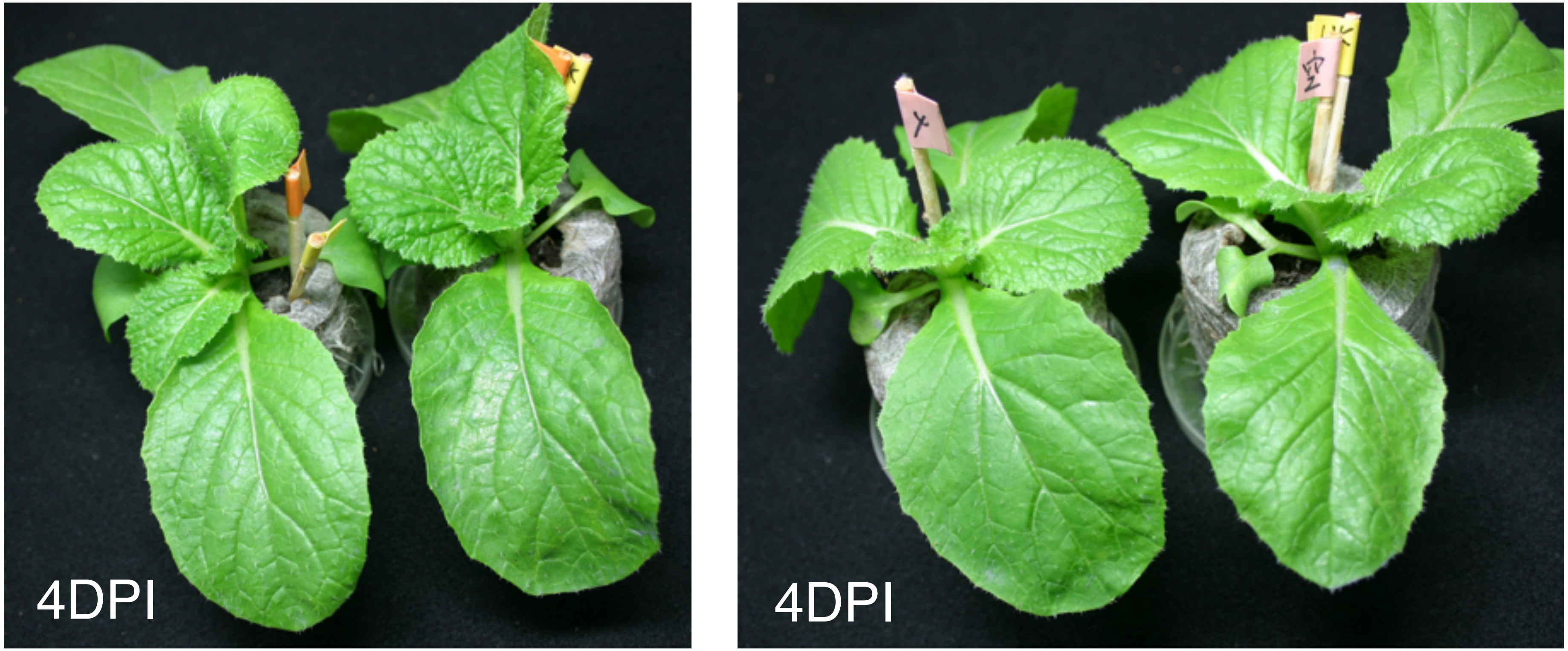

Mock TuMV-UK1

Aki-masari
(*Rnt1-1/rnt1-3*)

Mock TuMV-UK1

Ku-kai 65
(*Rnt1-1/ -*)

Inoculated leaves

Uninoculated upper leaves

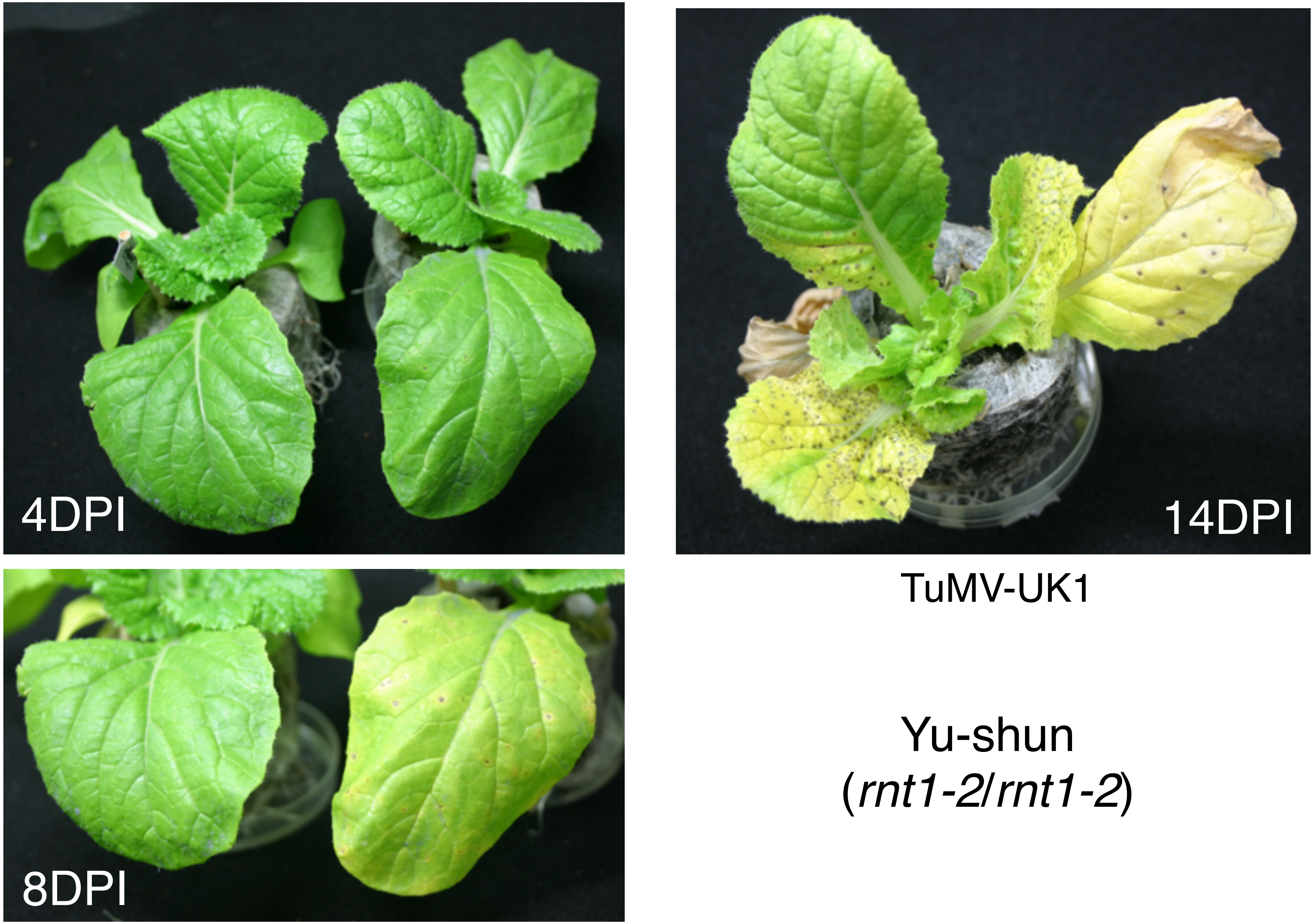

Mock TuMV-UK1

TuMV-UK1

Yu-shun
(*rnt1-2*/*rnt1-2*)

Inoculated leaves

Uninoculated upper leaves

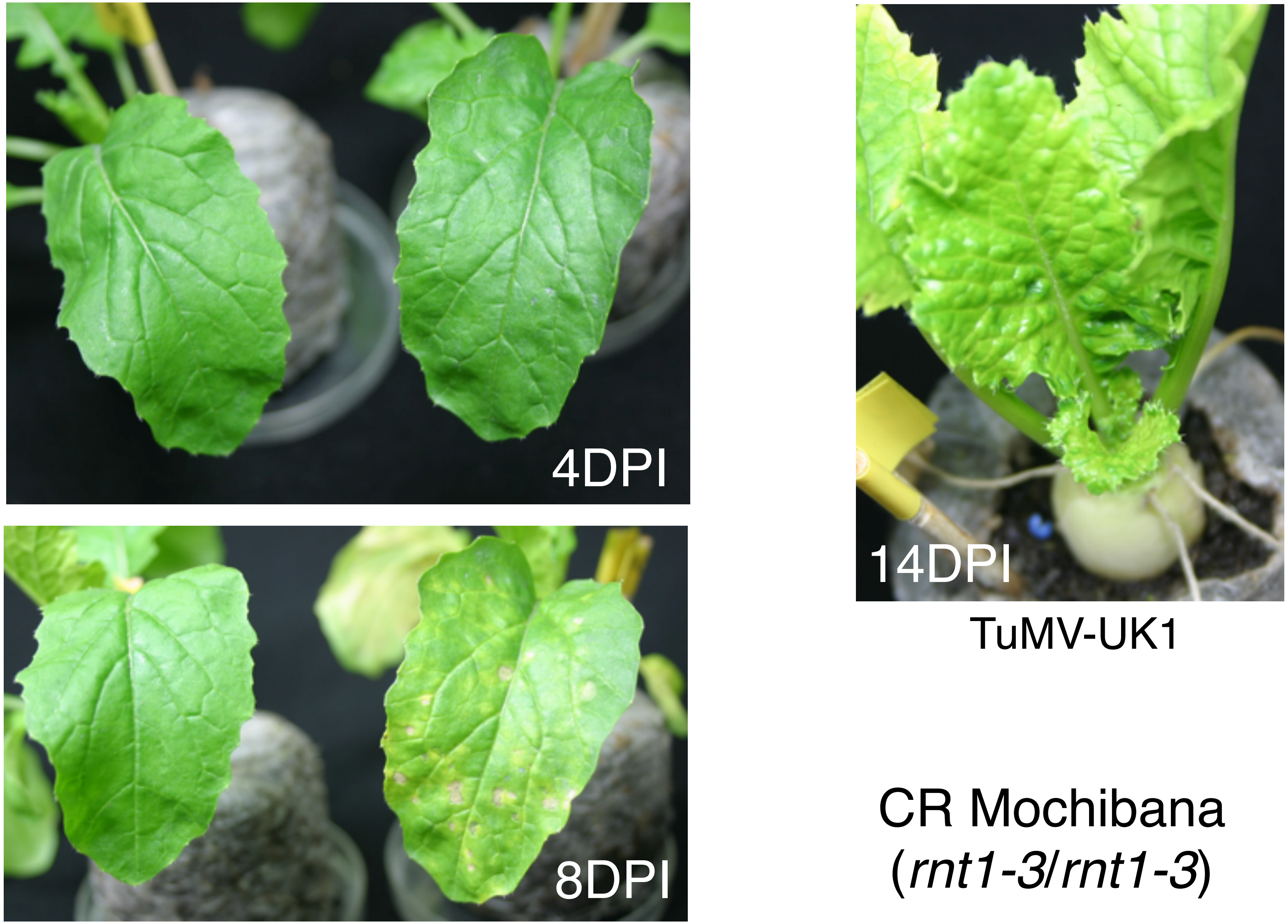

Mock

TuMV-UK1

TuMV-UK1

CR Mochibana
(*rnt1-3*/*rnt1-3*)

Inoculated leaves

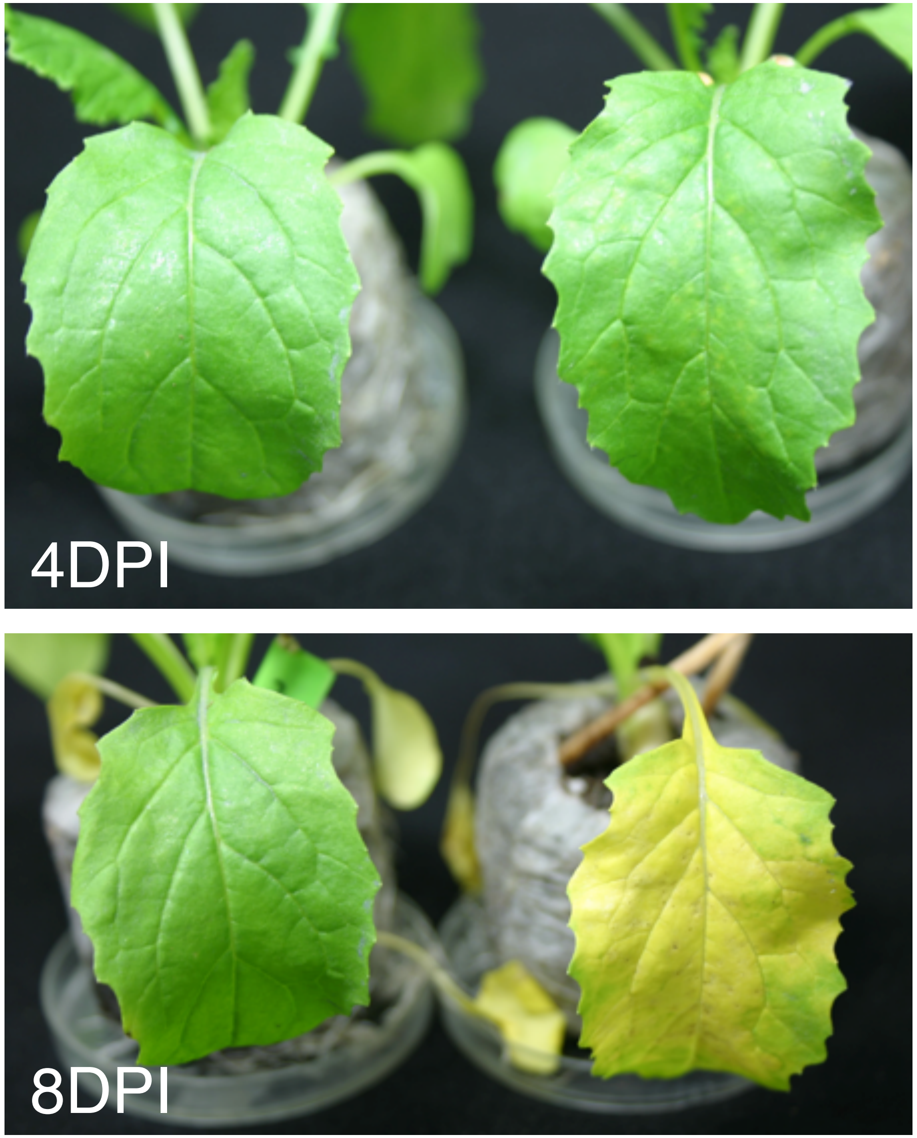

Mock TuMV-UK1

Uninoculated upper leaves

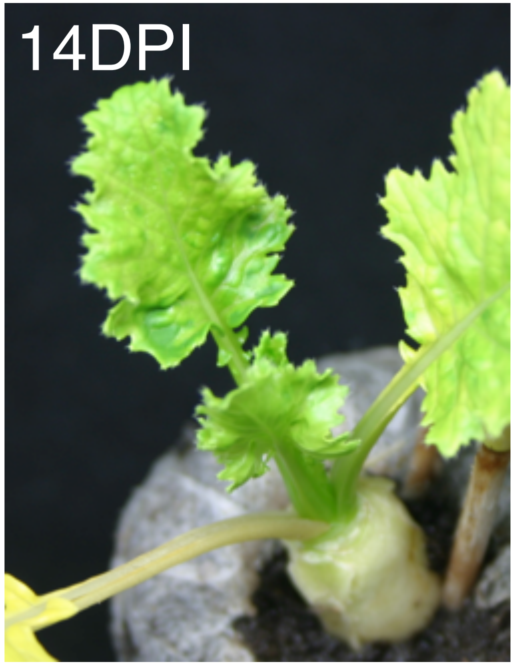

TuMV-UK1

Yukihime-kabu
(*rnt1-3*/*rnt1-3*)

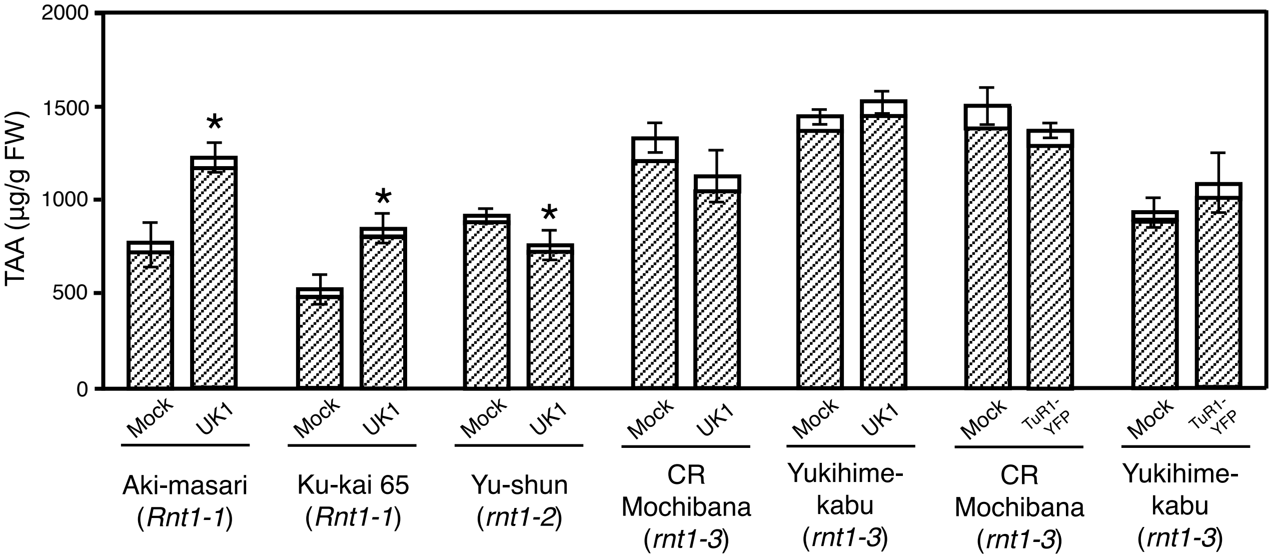

Supplementary Figure S5

Mean TAA (ascorbic acid [AS] and dehydroascorbic acid [DHA]) content in the five Chinese cabbage (*Brassica rapa* subsp. *pekinensis*) and turnip (*Brassica rapa* subsp. *rapa*) cultivars carrying different alleles at the *Rnt1* locus after inoculation with *Turnip mosaic virus* strains UK1 or TuR1-YFP. TAA content in the inoculated second true leaves was quantified 3 or 4 days after inoculation. The genotype at the *Rnt1* locus in each cultivar is described in parentheses. Hatched boxes, AS; white boxes, DHA. Error bars indicate standard error for biological triplicates for TAA content. An asterisk represents a significant difference determined by Student's *t*-test (**P* ≤ 0.05). FW, fresh mass.

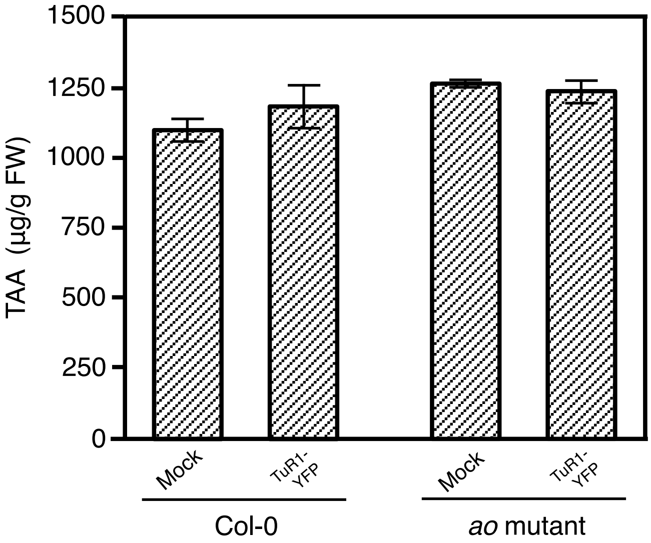

Supplementary Figure S6

Mean TAA (ascorbic acid [AS] and dehydroascorbic acid [DHA]) content in the *Arabidopsis thaliana* ecotype Col-0 and the *ao* mutant after inoculation with *Turnip mosaic virus* strain TuR1-YFP. TAA content in non-inoculated upper leaves was quantified 10 days after inoculation. Error bars indicate standard error for biological triplicates for TAA content. FW, fresh mass.

Supplementary Figure S7

Expression profiles determined with quantitative RT-PCR for genes for ascorbic acid (AS) synthesis, oxidation and recycling in turnip (*Brassica rapa* subsp. *rapa*) cvr. (A) Yukihime-kabu infected with *Turnip mosaic virus* (TuMV) strain UK1 at 4 days post inoculation (DPI) and (B) CR Mochibana infected with TuMV strain TuR1-YFP at 3 DPI. Asterisks represent significant differences determined by Student's t-test (*$P \leq 0.05$; ** $P \leq 0.01$). See Table S1 and Fig. S1 for full name of each gene and locations within the AS synthesis, oxidation and recycling pathways.

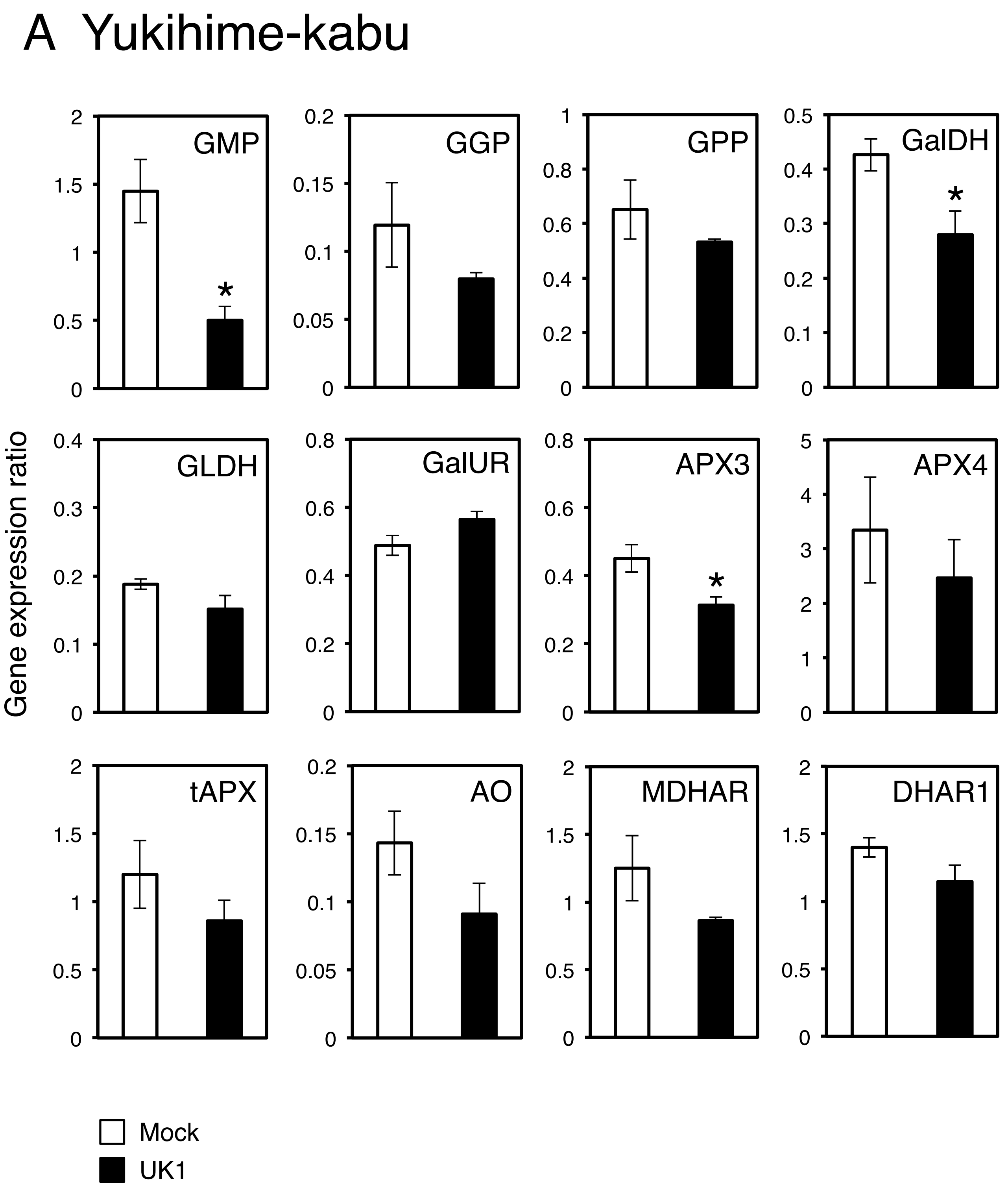

A Yukihime-kabu
Gene expression ratio
GMP
GGP
GPP
GalDH
GLDH
GalUR
APX3
APX4
tAPX
AO
MDHAR
DHAR1
*
0
0.5
1
1.5
2
0.05
0.1
0.15
0.2
0.4
0.6
0.8
0.3
0.1
3
4
5
Mock
UK1

B CR Mochibana

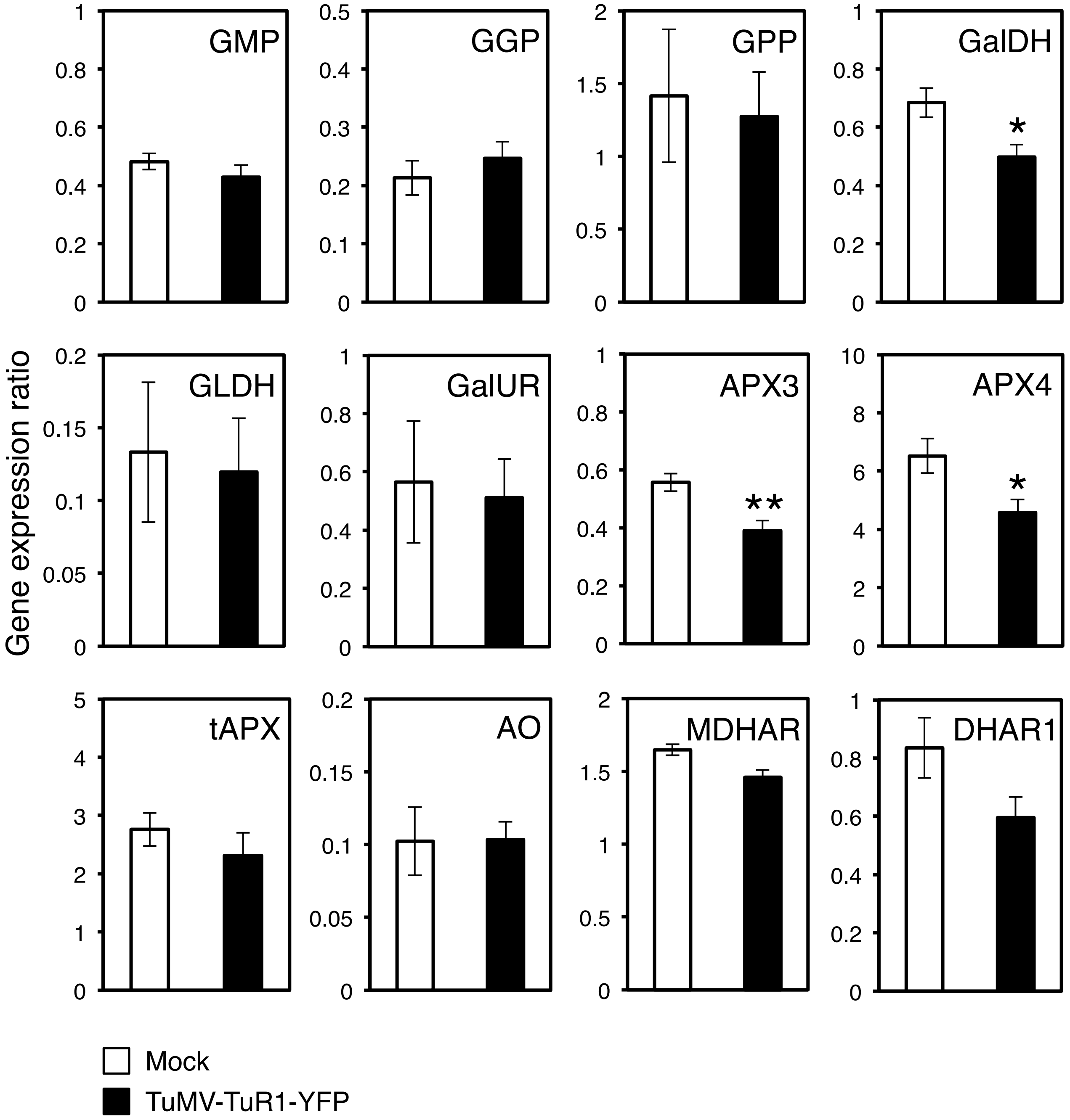

Supplementary Figure S8

Mean TAA [ascorbic acid (AS) and dehydroascorbic acid (DHA)] content in second true leaves of Chinese cabbage (*Brassica rapa* subsp. *pekinensis*) cv. Aki-masari at 24 h post treatment (HPT) with H_2O_2, salicylic acid (SA), methyl-jasmonate (MeJA) or abscisic acid (ABA) treatments. In the H_2O_2 treatment, TAA levels were also quantified at 1, 6 and 12 HPT. Hatched boxes, AS; white boxes, DHA. Error bars indicate standard error for biological triplicates for TAA content. An asterisk represents a significant difference determined by the Student's *t*-test (*$P \leq 0.05$); FW, fresh mass.

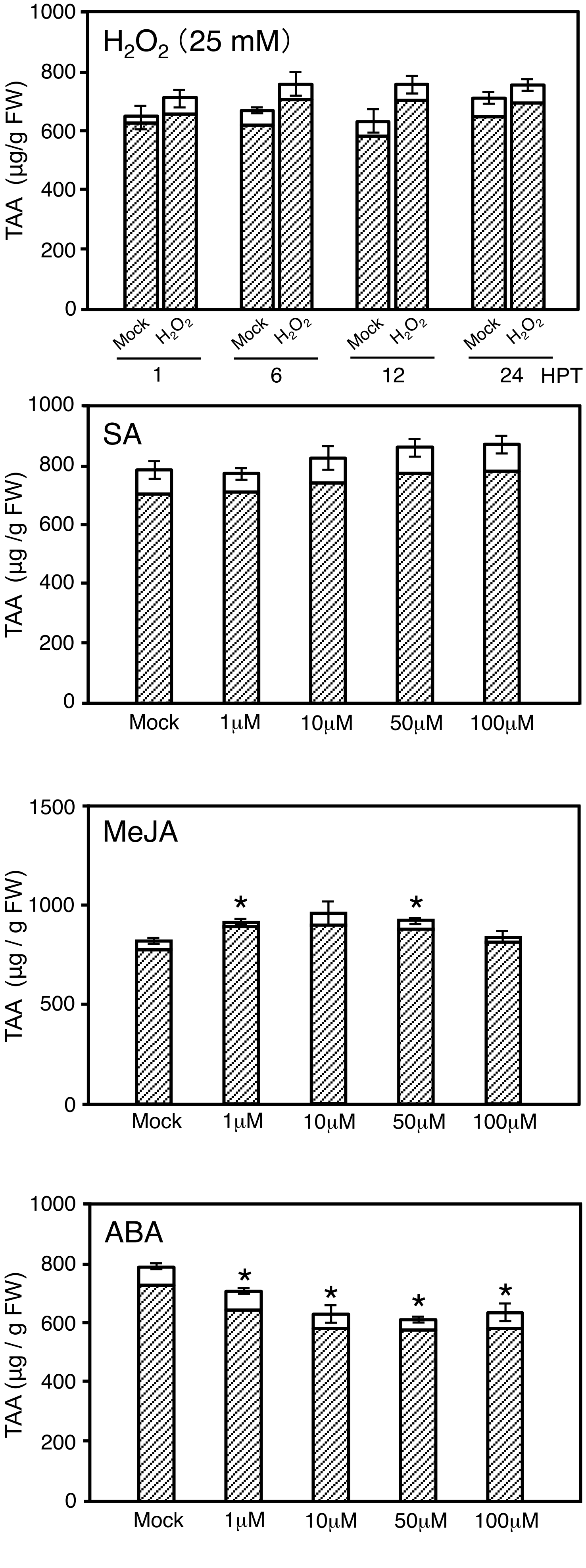

H2O2 (25 mM)
TAA (µg/g FW)
1000
800
600
400
200
0
Mock
H2O2
1
6
12
24
HPT
SA
TAA (µg /g FW)
Mock
1µM
10µM
50µM
100µM
MeJA
TAA (µg / g FW)
1500
1000
500
*
ABA
TAA (µg / g FW)

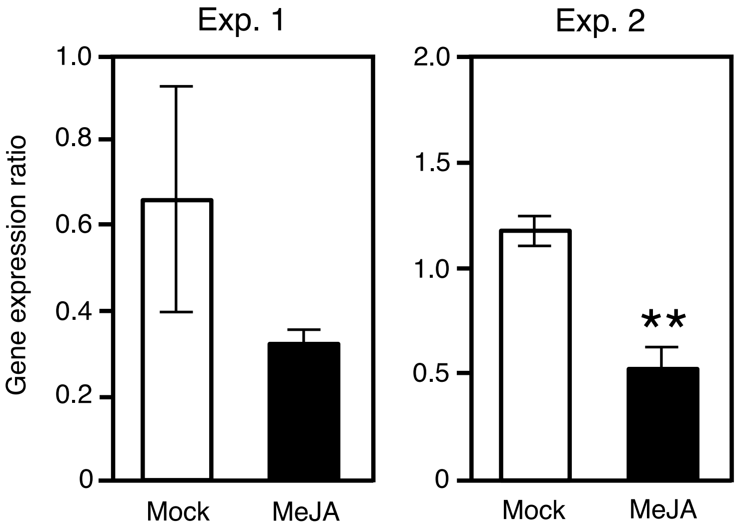

Supplementary Figure S9

Expression change of *PDF1.2* in Chinese cabbage (*Brassica rapa* subsp. *pekinensis*) cv. Akimasari after treatment with 50 μM methyl-jasmonate (MeJA). Transcript levels were determined by quantitative RT-PCR 24 h after treatment. Error bars indicate standard error for biological triplicates. An asterisk represents a significant difference determined by Student's t-test (**$P \leq 0.01$).

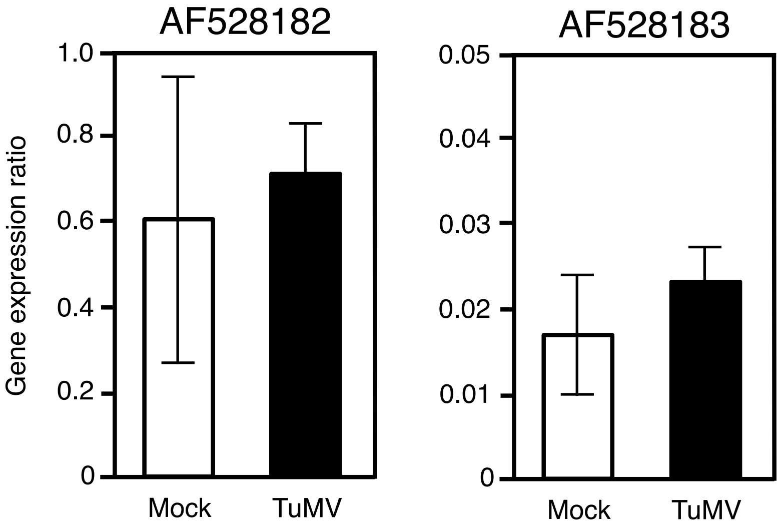

Supplementary Figure S10

Expression levels of the AF528182 and AF528183 genes in turnip (*Brassica rapa* subsp. *rapa*) cv. CR Mochibana by infection of *Turnip mosaic virus* (TuMV) strain TuR1-YFP. Transcript levels were determined by quantitative RT-PCR 3 days after inoculation. Error bars indicate standard error for biological triplicates.

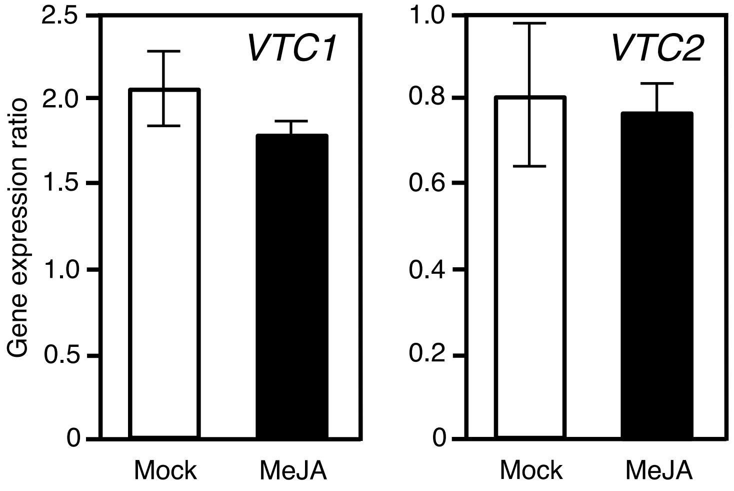

Supplementary Figure S11
Expression levels of the *VTC1* and *VTC2* genes in Chinese cabbage (*Brassica rapa* subsp. *pekinensis*) cv. Aki-masari after treatment with 50 μM methyl-jasmonate (MeJA). Transcript levels were determined by quantitative RT-PCR 24 h after treatment. Error bars indicate standard error for biological triplicates.
